# Supplementary material for: The extent to which genetics and lean grade affect fatty acid profiles and volatile compounds in organic pork
Source: PeerJ. 2019 Jul 17;7:e7322. doi: 10.7717/peerj.7322 (PMC6642625; doi:10.7717/peerj.7322)
Supplement: Supplemental Information 1 [file peerj-07-7322-s001.pdf]

Fit Group

Response perc_lipolysis

Whole Model

Effect Summary

Source	LogWorth	PValue
clasif	0,329	0,46854
genetictype	0,066	0,85862
gender	0,006	0,98687

Lack Of Fit

Source	DF	Sum of Squares	Mean Square	F Ratio
Lack Of Fit	2	144,5368	72,268	0,1780
Pure Error	17	6902,4595	406,027	**Prob > F**
Total Error	19	7046,9963		0,8385
				**Max RSq**
				0,0564

Summary of Fit

RSquare	0,036664
RSquare Adj	-0,11544
Root Mean Square Error	19,25862
Mean of Response	76,70232
Observations (or Sum Wgts)	23

Analysis of Variance

Source	DF	Sum of Squares	Mean Square	F Ratio
Model	3	268,2078	89,403	0,2410
Error	19	7046,9963	370,895	**Prob > F**
C. Total	22	7315,2041		0,8666

Parameter Estimates

Term	Estimate	Std Error	t Ratio	Prob>|t|
Intercept	76,867787	4,420876	17,39	<,0001*
clasif[less60]	-3,626814	4,903215	-0,74	0,4685
gender[female]	-0,098256	5,890837	-0,02	0,9869
genetictype[black]	0,8843439	4,89755	0,18	0,8586

Effect Tests

Source	Nparm	DF	Sum of Squares	F Ratio	Prob > F
clasif	1	1	202,92672	0,5471	0,4685
gender	1	1	0,10318	0,0003	0,9869
genetictype	1	1	12,09303	0,0326	0,8586

clasif

Least Squares Means Table

Level	Least Sq Mean	Std Error	Mean
less60	73,240972	6,9780935	73,8003
S	80,494601	6,2030261	80,4749

gender

Least Squares Means Table

Level	Least Sq Mean	Std Error	Mean
female	76,769531	8,3541954	78,9811
male	76,966043	6,2209023	75,7054

genetictype

Least Squares Means Table

Level	Least Sq Mean	Std Error	Mean
black	77,752131	6,2342758	76,6152
white	75,983443	6,9421957	76,8656

Fit Group

Response perc_proteolysis

Whole Model

Effect Summary

Source	LogWorth	PValue
genetictype	0,719	0,19107
gender	0,621	0,23934
clasif	0,225	0,59560

Lack Of Fit

Source	DF	Sum of Squares	Mean Square	F Ratio
Lack Of Fit	2	26,69434	13,3472	0,7539
Pure Error	17	300,96061	17,7036	**Prob > F**
Total Error	19	327,65495		0,4856
				**Max RSq**
				0,1821

Summary of Fit

RSquare	0,109533
RSquare Adj	-0,03107
Root Mean Square Error	4,15271
Mean of Response	5,360806
Observations (or Sum Wgts)	23

Analysis of Variance

Source	DF	Sum of Squares	Mean Square	F Ratio
Model	3	40,30350	13,4345	0,7790
Error	19	327,65495	17,2450	**Prob > F**
C. Total	22	367,95845		0,5202

Parameter Estimates

Term	Estimate	Std Error	t Ratio	Prob>|t|
Intercept	5,4544027	0,953267	5,72	<,0001*
clasif[less60]	0,5707291	1,057273	0,54	0,5956
gender[female]	1,5430314	1,270233	1,21	0,2393
genetictype[black]	1,4317685	1,056052	1,36	0,1911

Effect Tests

Source	Nparm	DF	Sum of Squares	F Ratio	Prob > F
clasif	1	1	5,025146	0,2914	0,5956
gender	1	1	25,447550	1,4756	0,2393
genetictype	1	1	31,698473	1,8381	0,1911

clasif

Least Squares Means Table

Level	Least Sq Mean	Std Error	Mean
less60	6,0251318	1,5046765	5,49044
S	4,8836735	1,3375498	5,19228

gender

Least Squares Means Table

Level	Least Sq Mean	Std Error	Mean
female	6,9974341	1,8014034	5,97616
male	3,9113712	1,3414044	5,09159

genetictype

Least Squares Means Table

Level	Least Sq Mean	Std Error	Mean
black	6,8861712	1,3442881	5,94486
white	4,0226341	1,4969359	4,26571

Fit Group

Response perc_microbial

Whole Model

Effect Summary

Source	LogWorth	PValue
clasif	0,389	0,40825
genetictype	0,190	0,64555
gender	0,064	0,86201

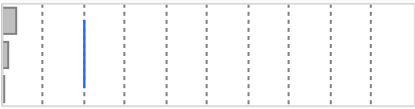

Lack Of Fit

Source	DF	Sum of Squares	Mean Square	F Ratio
Lack Of Fit	2	130,9965	65,498	0,1510
Pure Error	17	7375,9537	433,880	**Prob > F**
Total Error	19	7506,9502		0,8610
				**Max RSq**
				0,0772

Summary of Fit

RSquare	0,060845
RSquare Adj	-0,08744
Root Mean Square Error	19,87719
Mean of Response	14,94762
Observations (or Sum Wgts)	23

Analysis of Variance

Source	DF	Sum of Squares	Mean Square	F Ratio
Model	3	486,3521	162,117	0,4103
Error	19	7506,9502	395,103	**Prob > F**
C. Total	22	7993,3023		0,7474

Parameter Estimates

Term	Estimate	Std Error	t Ratio	Prob>|t|
Intercept	14,689234	4,56287	3,22	0,0045*
clasif[less60]	4,2797661	5,060701	0,85	0,4083
gender[female]	-1,071218	6,080044	-0,18	0,8620
genetictype[black]	-2,362482	5,054854	-0,47	0,6456

Effect Tests

Source	Nparm	DF	Sum of Squares	F Ratio	Prob > F
clasif	1	1	282,57168	0,7152	0,4083
gender	1	1	12,26458	0,0310	0,8620
genetictype	1	1	86,30374	0,2184	0,6456

clasif

Least Squares Means Table

Level	Least Sq Mean	Std Error	Mean
less60	18,969000	7,2022225	18,6033
S	10,409468	6,4022607	10,1952

gender

Least Squares Means Table

Level	Least Sq Mean	Std Error	Mean
female	13,618015	8,6225233	11,5735
male	15,760452	6,4207110	16,4238

genetictype

Least Squares Means Table

Level	Least Sq Mean	Std Error	Mean
black	12,326752	6,4345141	14,5389
white	17,051715	7,1651716	15,7140

Fit Group

Response perc_unknown

Whole Model

Effect Summary

Source	LogWorth	PValue
clasif	2,040	0,00912
gender	0,328	0,46974
genetictype	0,039	0,91346

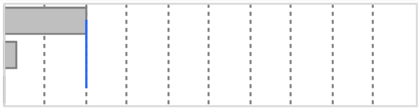

Lack Of Fit

Source	DF	Sum of Squares	Mean Square	F Ratio
Lack Of Fit	2	0,214719	0,10736	0,0352
Pure Error	17	51,864630	3,05086	**Prob > F**
Total Error	19	52,079349		0,9655
				**Max RSq**
				0,3312

Summary of Fit

RSquare	0,328437
RSquare Adj	0,2224
Root Mean Square Error	1,655602
Mean of Response	2,989253
Observations (or Sum Wgts)	23

Analysis of Variance

Source	DF	Sum of Squares	Mean Square	F Ratio
Model	3	25,470060	8,49002	3,0974
Error	19	52,079349	2,74102	**Prob > F**
C. Total	22	77,549409		0,0514

Parameter Estimates

Term	Estimate	Std Error	t Ratio	Prob>|t|
Intercept	2,9885771	0,380049	7,86	<,0001*
clasif[less60]	-1,223681	0,421514	-2,90	0,0091*
gender[female]	-0,373557	0,506416	-0,74	0,4697
genetictype[black]	0,0463692	0,421027	0,11	0,9135

Effect Tests

Source	Nparm	DF	Sum of Squares	F Ratio	Prob > F
clasif	1	1	23,100687	8,4278	0,0091*
gender	1	1	1,491456	0,5441	0,4697
genetictype	1	1	0,033247	0,0121	0,9135

clasif

Least Squares Means Table

Level	Least Sq Mean	Std Error	Mean
less60	1,7648962	0,59988436	2,10595
S	4,2122579	0,53325429	4,13755

gender

Least Squares Means Table

Level	Least Sq Mean	Std Error	Mean
female	2,6150200	0,71818343	3,46921
male	3,3621341	0,53479105	2,77927

genetictype

Least Squares Means Table

Level	Least Sq Mean	Std Error	Mean
black	3,0349462	0,53594073	2,90099
white	2,9422079	0,59679834	3,15474

Fit Group

Response Methanethiol

Whole Model

Effect Summary

Source	LogWorth	PValue
clasif	0,490	0,32338
genetictype	0,317	0,48231
gender	0,173	0,67126

Lack Of Fit

Source	DF	Sum of Squares	Mean Square	F Ratio
Lack Of Fit	2	2,46014	1,2301	0,0548
Pure Error	17	381,84463	22,4614	**Prob > F**
Total Error	19	384,30477		0,9469
				**Max RSq**
				0,1128

Summary of Fit

RSquare	0,107037
RSquare Adj	-0,03396
Root Mean Square Error	4,497396
Mean of Response	2,381032
Observations (or Sum Wgts)	23

Analysis of Variance

Source	DF	Sum of Squares	Mean Square	F Ratio
Model	3	46,06570	15,3552	0,7592
Error	19	384,30477	20,2266	**Prob > F**
C. Total	22	430,37047		0,5308

Parameter Estimates

Term	Estimate	Std Error	t Ratio	Prob>|t|
Intercept	2,2470027	1,032391	2,18	0,0423*
clasif[less60]	1,1609203	1,14503	1,01	0,3234
gender[female]	-0,593032	1,375666	-0,43	0,6713
genetictype[black]	-0,819624	1,143707	-0,72	0,4823

Effect Tests

Source	Nparm	DF	Sum of Squares	F Ratio	Prob > F
clasif	1	1	20,791862	1,0279	0,3234
gender	1	1	3,758828	0,1858	0,6713
genetictype	1	1	10,387755	0,5136	0,4823

clasif

Least Squares Means Table

Level	Least Sq Mean	Std Error	Mean
less60	3,4079230	1,6295686	3,46838
S	1,0860824	1,4485700	0,96748

gender

Least Squares Means Table

Level	Least Sq Mean	Std Error	Mean
female	1,6539709	1,9509246	1,17601
male	2,8400345	1,4527445	2,90823

genetictype

Least Squares Means Table

Level	Least Sq Mean	Std Error	Mean
black	1,4273790	1,4558676	2,24924
white	3,0666264	1,6211855	2,62814

Fit Group

Response Pentane

Whole Model

Effect Summary

Source	LogWorth	PValue
genetictype	0,210	0,61707
gender	0,013	0,97071
clasif	0,006	0,98590

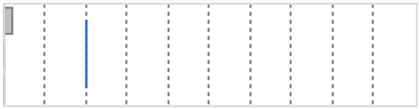

Lack Of Fit

Source	DF	Sum of Squares	Mean Square	F Ratio
Lack Of Fit	2	8957,44	4478,7	0,3630
Pure Error	17	209725,21	12336,8	**Prob > F**
Total Error	19	218682,64		0,7008
				**Max RSq**
				0,0572

Summary of Fit

RSquare	0,016945
RSquare Adj	-0,13827
Root Mean Square Error	107,2829
Mean of Response	99,54106
Observations (or Sum Wgts)	23

Analysis of Variance

Source	DF	Sum of Squares	Mean Square	F Ratio
Model	3	3769,49	1256,5	0,1092
Error	19	218682,64	11509,6	**Prob > F**
C. Total	22	222452,13		0,9537

Parameter Estimates

Term	Estimate	Std Error	t Ratio	Prob>|t|
Intercept	95,7341	24,62711	3,89	0,0010*
clasif[less60]	0,4891698	27,31405	0,02	0,9859
gender[female]	1,2209263	32,81573	0,04	0,9707
genetictype[black]	13,868691	27,28249	0,51	0,6171

Effect Tests

Source	Nparm	DF	Sum of Squares	F Ratio	Prob > F
clasif	1	1	3,6915	0,0003	0,9859
gender	1	1	15,9322	0,0014	0,9707
genetictype	1	1	2974,1555	0,2584	0,6171

clasif

Least Squares Means Table

Level	Least Sq Mean	Std Error	Mean
less60	96,223269	38,872451	102,658
S	95,244930	34,554829	95,489

gender

Least Squares Means Table

Level	Least Sq Mean	Std Error	Mean
female	96,955026	46,538220	90,662
male	94,513173	34,654410	103,426

genetictype

Least Squares Means Table

Level	Least Sq Mean	Std Error	Mean
black	109,60279	34,728909	108,871
white	81,86541	38,672477	82,048

Fit Group

Response Propanal

Whole Model

Effect Summary

Source	LogWorth	PValue
gender	0,300	0,50092
clasif	0,119	0,75957
genetictype	0,001	0,99858

Lack Of Fit

Source	DF	Sum of Squares	Mean Square	F Ratio
Lack Of Fit	2	8382,771	4191,39	2,6432
Pure Error	17	26957,409	1585,73	**Prob > F**
Total Error	19	35340,180		0,1001
				**Max RSq**
				0,2608

Summary of Fit

RSquare	0,030875
RSquare Adj	-0,12215
Root Mean Square Error	43,12783
Mean of Response	65,0226
Observations (or Sum Wgts)	23

Analysis of Variance

Source	DF	Sum of Squares	Mean Square	F Ratio
Model	3	1125,875	375,29	0,2018
Error	19	35340,180	1860,01	**Prob > F**
C. Total	22	36466,054		0,8939

Parameter Estimates

Term	Estimate	Std Error	t Ratio	Prob>|t|
Intercept	68,113745	9,900125	6,88	<,0001*
clasif[less60]	3,409267	10,98028	0,31	0,7596
gender[female]	9,0513994	13,19196	0,69	0,5009
genetictype[black]	0,0197891	10,96759	0,00	0,9986

Effect Tests

Source	Nparm	DF	Sum of Squares	F Ratio	Prob > F
clasif	1	1	179,31252	0,0964	0,7596
gender	1	1	875,64466	0,4708	0,5009
genetictype	1	1	0,00606	0,0000	0,9986

clasif

Least Squares Means Table

Level	Least Sq Mean	Std Error	Mean
less60	71,523012	15,626767	63,8748
S	64,704478	13,891078	66,5148

gender

Least Squares Means Table

Level	Least Sq Mean	Std Error	Mean
female	77,165144	18,708415	74,7215
male	59,062345	13,931110	60,7793

genetictype

Least Squares Means Table

Level	Least Sq Mean	Std Error	Mean
black	68,133534	13,961059	62,6323
white	68,093956	15,546377	69,5045

Fit Group

Response Acetone

Whole Model

Effect Summary

Source	LogWorth	PValue
gender	0,781	0,16558
clasif	0,448	0,35644
genetictype	0,040	0,91278

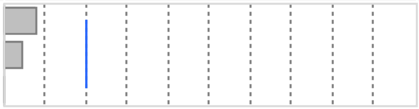

Lack Of Fit

Source	DF	Sum of Squares	Mean Square	F Ratio
Lack Of Fit	2	4643,762	2321,88	1,1697
Pure Error	17	33745,356	1985,02	**Prob > F**
Total Error	19	38389,117		0,3342
				**Max RSq**
				0,2207

Summary of Fit

RSquare	0,113479
RSquare Adj	-0,0265
Root Mean Square Error	44,94975
Mean of Response	58,52873
Observations (or Sum Wgts)	23

Analysis of Variance

Source	DF	Sum of Squares	Mean Square	F Ratio
Model	3	4913,978	1637,99	0,8107
Error	19	38389,117	2020,48	**Prob > F**
C. Total	22	43303,095		0,5036

Parameter Estimates

Term	Estimate	Std Error	t Ratio	Prob>|t|
Intercept	64,48988	10,31835	6,25	<,0001*
clasif[less60]	10,81663	11,44414	0,95	0,3564
gender[female]	19,826462	13,74925	1,44	0,1656
genetictype[black]	1,2688169	11,43091	0,11	0,9128

Effect Tests

Source	Nparm	DF	Sum of Squares	F Ratio	Prob > F
clasif	1	1	1804,9804	0,8933	0,3564
gender	1	1	4201,3308	2,0794	0,1656
genetictype	1	1	24,8938	0,0123	0,9128

clasif

Least Squares Means Table

Level	Least Sq Mean	Std Error	Mean
less60	75,306510	16,286915	59,2135
S	53,673250	14,477903	57,6385

gender

Least Squares Means Table

Level	Least Sq Mean	Std Error	Mean
female	84,316342	19,498746	76,0464
male	44,663419	14,519626	50,8647

genetictype

Least Squares Means Table

Level	Least Sq Mean	Std Error	Mean
black	65,758697	14,550840	54,8248
white	63,221063	16,203129	65,4735

Fit Group

Response Carbon disulfide

Whole Model

Effect Summary

Source	LogWorth	PValue
genetictype	0,203	0,62590
clasif	0,096	0,80102
gender	0,012	0,97278

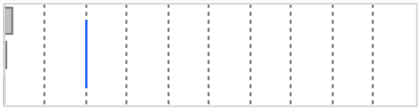

Lack Of Fit

Source	DF	Sum of Squares	Mean Square	F Ratio
Lack Of Fit	2	8,03465	4,0173	0,1389
Pure Error	17	491,61962	28,9188	**Prob > F**
Total Error	19	499,65426		0,8713
				**Max RSq**
				0,0323

Summary of Fit

RSquare	0,016483
RSquare Adj	-0,13881
Root Mean Square Error	5,128118
Mean of Response	10,46312
Observations (or Sum Wgts)	23

Analysis of Variance

Source	DF	Sum of Squares	Mean Square	F Ratio
Model	3	8,37397	2,7913	0,1061
Error	19	499,65426	26,2976	**Prob > F**
C. Total	22	508,02823		0,9555

Parameter Estimates

Term	Estimate	Std Error	t Ratio	Prob>|t|
Intercept	10,59506	1,177175	9,00	<,0001*
clasif[less60]	0,3336943	1,305611	0,26	0,8010
gender[female]	-0,05423	1,568591	-0,03	0,9728
genetictype[black]	-0,646237	1,304102	-0,50	0,6259

Effect Tests

Source	Nparm	DF	Sum of Squares	F Ratio	Prob > F
clasif	1	1	1,7178537	0,0653	0,8010
gender	1	1	0,0314320	0,0012	0,9728
genetictype	1	1	6,4576750	0,2456	0,6259

clasif

Least Squares Means Table

Level	Least Sq Mean	Std Error	Mean
less60	10,928754	1,8581021	10,6267
S	10,261365	1,6517198	10,2505

gender

Least Squares Means Table

Level	Least Sq Mean	Std Error	Mean
female	10,540830	2,2245256	10,5794
male	10,649289	1,6564798	10,4122

genetictype

Least Squares Means Table

Level	Least Sq Mean	Std Error	Mean
black	9,948823	1,6600409	10,0998
white	11,241297	1,8485433	11,1443

Fit Group

Response n-Hexane

Whole Model

Effect Summary

Source	LogWorth	PValue
gender	0,413	0,38655
genetictype	0,177	0,66474
clasif	0,074	0,84412

Lack Of Fit

Source	DF	Sum of Squares	Mean Square	F Ratio
Lack Of Fit	2	1772,462	886,23	0,1786
Pure Error	17	84340,480	4961,20	**Prob > F**
Total Error	19	86112,943		0,8380
				**Max RSq**
				0,1452

Summary of Fit

RSquare	0,127187
RSquare Adj	-0,01063
Root Mean Square Error	67,32206
Mean of Response	58,50653
Observations (or Sum Wgts)	23

Analysis of Variance

Source	DF	Sum of Squares	Mean Square	F Ratio
Model	3	12548,430	4182,81	0,9229
Error	19	86112,943	4532,26	**Prob > F**
C. Total	22	98661,373		0,4487

Parameter Estimates

Term	Estimate	Std Error	t Ratio	Prob>|t|
Intercept	68,387537	15,45399	4,43	0,0003*
clasif[less60]	-3,416649	17,14009	-0,20	0,8441
gender[female]	18,250546	20,59251	0,89	0,3866
genetictype[black]	-7,536891	17,12029	-0,44	0,6647

Effect Tests

Source	Nparm	DF	Sum of Squares	F Ratio	Prob > F
clasif	1	1	180,0899	0,0397	0,8441
gender	1	1	3559,9849	0,7855	0,3866
genetictype	1	1	878,3693	0,1938	0,6647

clasif

Least Squares Means Table

Level	Least Sq Mean	Std Error	Mean
less60	64,970888	24,393211	45,4698
S	71,804187	21,683820	75,4543

gender

Least Squares Means Table

Level	Least Sq Mean	Std Error	Mean
female	86,638083	29,203629	92,3086
male	50,136992	21,746310	43,7181

genetictype

Least Squares Means Table

Level	Least Sq Mean	Std Error	Mean
black	60,850646	21,793059	46,3280
white	75,924428	24,267724	81,3412

Fit Group

Response Acetic acid ethenyl ester

Whole Model

Effect Summary

Source	LogWorth	PValue
clasif	0,587	0,25861
genetictype	0,461	0,34583
gender	0,211	0,61451

Lack Of Fit

Source	DF	Sum of Squares	Mean Square	F Ratio
Lack Of Fit	2	1192,075	596,04	0,1299
Pure Error	16	73423,734	4588,98	**Prob > F**
Total Error	18	74615,809		0,8791
				**Max RSq**
				0,1488

Summary of Fit

RSquare	0,134956
RSquare Adj	-0,00922
Root Mean Square Error	64,38418
Mean of Response	43,00786
Observations (or Sum Wgts)	22

Analysis of Variance

Source	DF	Sum of Squares	Mean Square	F Ratio
Model	3	11640,889	3880,30	0,9361
Error	18	74615,809	4145,32	**Prob > F**
C. Total	21	86256,698		0,4437

Parameter Estimates

Term	Estimate	Std Error	t Ratio	Prob>|t|
Intercept	43,846819	15,15129	2,89	0,0097*
clasif[less60]	19,315301	16,55756	1,17	0,2586
gender[female]	-10,41433	20,31947	-0,51	0,6145
genetictype[black]	-17,55031	18,12879	-0,97	0,3458

Effect Tests

Source	Nparm	DF	Sum of Squares	F Ratio	Prob > F
clasif	1	1	5641,1624	1,3608	0,2586
gender	1	1	1088,9218	0,2627	0,6145
genetictype	1	1	3884,9926	0,9372	0,3458

clasif

Least Squares Means Table

Level	Least Sq Mean	Std Error	Mean
less60	63,162120	24,007947	60,1405
S	24,531518	20,761683	22,4487

gender

Least Squares Means Table

Level	Least Sq Mean	Std Error	Mean
female	33,432486	27,978955	27,1574
male	54,261153	22,406762	50,4047

genetictype

Least Squares Means Table

Level	Least Sq Mean	Std Error	Mean
black	26,296510	21,311237	40,3721
white	61,397129	25,734427	48,6559

Fit Group

Response 2-Butanone

Whole Model

Effect Summary

Source	LogWorth	PValue
clasif	1,242	0,05732
gender	0,411	0,38790
genetictype	0,325	0,47308

Lack Of Fit

Source	DF	Sum of Squares	Mean Square	F Ratio
Lack Of Fit	2	58,3821	29,1911	0,4214
Pure Error	17	1177,4915	69,2642	**Prob > F**
Total Error	19	1235,8737		0,6628
				**Max RSq**
				0,2454

Summary of Fit

RSquare	0,207945
RSquare Adj	0,082884
Root Mean Square Error	8,065109
Mean of Response	7,429741
Observations (or Sum Wgts)	23

Analysis of Variance

Source	DF	Sum of Squares	Mean Square	F Ratio
Model	3	324,4644	108,155	1,6627
Error	19	1235,8737	65,046	**Prob > F**
C. Total	22	1560,3380		0,2086

Parameter Estimates

Term	Estimate	Std Error	t Ratio	Prob>|t|
Intercept	7,2839124	1,85137	3,93	0,0009*
clasif[less60]	4,1550117	2,053364	2,02	0,0573
gender[female]	2,1800765	2,466959	0,88	0,3879
genetictype[black]	1,501388	2,050992	0,73	0,4731

Effect Tests

Source	Nparm	DF	Sum of Squares	F Ratio	Prob > F
clasif	1	1	266,33796	4,0946	0,0573
gender	1	1	50,79721	0,7809	0,3879
genetictype	1	1	34,85608	0,5359	0,4731

clasif

Least Squares Means Table

Level	Least Sq Mean	Std Error	Mean
less60	11,438924	2,9222798	10,4027
S	3,128901	2,5976978	3,5649

gender

Least Squares Means Table

Level	Least Sq Mean	Std Error	Mean
female	9,4639889	3,4985626	5,85267
male	5,1038360	2,6051840	8,11971

genetictype

Least Squares Means Table

Level	Least Sq Mean	Std Error	Mean
black	8,7853005	2,6107845	8,57158
white	5,7825244	2,9072466	5,28879

Fit Group

Response Ethyl Acetate

Whole Model

Effect Summary

Source	LogWorth	PValue
clasif	0,376	0,42028
gender	0,096	0,80194
genetictype	0,093	0,80734

Lack Of Fit

Source	DF	Sum of Squares	Mean Square	F Ratio
Lack Of Fit	2	6551,69	3275,8	0,0937
Pure Error	17	594627,82	34978,1	**Prob > F**
Total Error	19	601179,51		0,9111
				**Max RSq**
				0,0752

Summary of Fit

RSquare	0,064961
RSquare Adj	-0,08268
Root Mean Square Error	177,8792
Mean of Response	58,85758
Observations (or Sum Wgts)	23

Analysis of Variance

Source	DF	Sum of Squares	Mean Square	F Ratio
Model	3	41766,15	13922,0	0,4400
Error	19	601179,51	31641,0	**Prob > F**
C. Total	22	642945,66		0,7270

Parameter Estimates

Term	Estimate	Std Error	t Ratio	Prob>|t|
Intercept	51,979836	40,83273	1,27	0,2184
clasif[less60]	37,307563	45,28778	0,82	0,4203
gender[female]	-13,84066	54,40979	-0,25	0,8019
genetictype[black]	-11,1858	45,23545	-0,25	0,8073

Effect Tests

Source	Nparm	DF	Sum of Squares	F Ratio	Prob > F
clasif	1	1	21472,485	0,6786	0,4203
gender	1	1	2047,436	0,0647	0,8019
genetictype	1	1	1934,758	0,0611	0,8073

clasif

Least Squares Means Table

Level	Least Sq Mean	Std Error	Mean
less60	89,287399	64,452067	94,9756
S	14,672274	57,293278	11,9041

gender

Least Squares Means Table

Level	Least Sq Mean	Std Error	Mean
female	38,139172	77,162216	16,2848
male	65,820501	57,458388	77,4832

genetictype

Least Squares Means Table

Level	Least Sq Mean	Std Error	Mean
black	40,794039	57,581911	63,3797
white	63,165633	64,120502	50,3786

Fit Group

Response 1-Propanol, 2-methyl-

Whole Model

Effect Summary

Source	LogWorth	PValue
genetictype	0,516	0,30510
clasif	0,176	0,66666
gender	0,054	0,88216

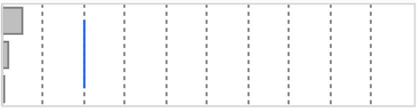

Lack Of Fit

Source	DF	Sum of Squares	Mean Square	F Ratio
Lack Of Fit	2	883,901	441,95	0,2903
Pure Error	17	25880,169	1522,36	**Prob > F**
Total Error	19	26764,071		0,7517
				**Max RSq**
				0,1077

Summary of Fit

RSquare	0,077189
RSquare Adj	-0,06852
Root Mean Square Error	37,53179
Mean of Response	24,65363
Observations (or Sum Wgts)	23

Analysis of Variance

Source	DF	Sum of Squares	Mean Square	F Ratio
Model	3	2238,684	746,23	0,5298
Error	19	26764,071	1408,64	**Prob > F**
C. Total	22	29002,754		0,6673

Parameter Estimates

Term	Estimate	Std Error	t Ratio	Prob>|t|
Intercept	21,462327	8,615538	2,49	0,0222*
clasif[less60]	-4,180881	9,555535	-0,44	0,6667
gender[female]	-1,724783	11,48024	-0,15	0,8822
genetictype[black]	10,059946	9,544494	1,05	0,3051

Effect Tests

Source	Nparm	DF	Sum of Squares	F Ratio	Prob > F
clasif	1	1	269,6647	0,1914	0,6667
gender	1	1	31,7955	0,0226	0,8822
genetictype	1	1	1564,8908	1,1109	0,3051

clasif

Least Squares Means Table

Level	Least Sq Mean	Std Error	Mean
less60	17,281446	13,599121	24,1578
S	25,643208	12,088646	25,2983

gender

Least Squares Means Table

Level	Least Sq Mean	Std Error	Mean
female	19,737544	16,280911	18,4125
male	23,187110	12,123484	27,3841

genetictype

Least Squares Means Table

Level	Least Sq Mean	Std Error	Mean
black	31,522273	12,149547	31,3935
white	11,402381	13,529163	12,0164

Fit Group

Response Butanal, 3-methyl-

Whole Model

Effect Summary

Source	LogWorth	PValue
genetictype	0,307	0,49310
clasif	0,250	0,56285
gender	0,234	0,58390

Lack Of Fit

Source	DF	Sum of Squares	Mean Square	F Ratio
Lack Of Fit	2	1500,201	750,10	0,6051
Pure Error	17	21072,815	1239,58	**Prob > F**
Total Error	19	22573,017		0,5574
				**Max RSq**
				0,1056

Summary of Fit

RSquare	0,041935
RSquare Adj	-0,10934
Root Mean Square Error	34,46815
Mean of Response	13,04464
Observations (or Sum Wgts)	23

Analysis of Variance

Source	DF	Sum of Squares	Mean Square	F Ratio
Model	3	988,035	329,34	0,2772
Error	19	22573,017	1188,05	**Prob > F**
C. Total	22	23561,051		0,8411

Parameter Estimates

Term	Estimate	Std Error	t Ratio	Prob>|t|
Intercept	12,804883	7,91227	1,62	0,1221
clasif[less60]	5,168144	8,775537	0,59	0,5628
gender[female]	5,8745109	10,54314	0,56	0,5839
genetictype[black]	6,125807	8,765398	0,70	0,4931

Effect Tests

Source	Nparm	DF	Sum of Squares	F Ratio	Prob > F
clasif	1	1	412,05746	0,3468	0,5628
gender	1	1	368,84158	0,3105	0,5839
genetictype	1	1	580,25558	0,4884	0,4931

clasif

Least Squares Means Table

Level	Least Sq Mean	Std Error	Mean
less60	17,973027	12,489055	16,3008
S	7,636739	11,101876	8,8116

gender

Least Squares Means Table

Level	Least Sq Mean	Std Error	Mean
female	18,679394	14,951935	12,3625
male	6,930372	11,133870	13,3431

genetictype

Least Squares Means Table

Level	Least Sq Mean	Std Error	Mean
black	18,930690	11,157806	16,3454
white	6,679076	12,424806	6,8557

Fit Group

Response Butanal, 2-methyl-

Whole Model

Effect Summary

Source	LogWorth	PValue
genetictype	0,537	0,29012
gender	0,428	0,37306
clasif	0,256	0,55449

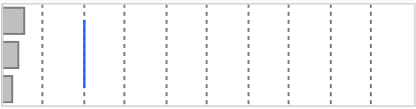

Lack Of Fit

Source	DF	Sum of Squares	Mean Square	F Ratio
Lack Of Fit	2	646,3541	323,177	1,4489
Pure Error	17	3791,7860	223,046	**Prob > F**
Total Error	19	4438,1401		0,2624
				**Max RSq**
				0,2107

Summary of Fit

RSquare	0,076136
RSquare Adj	-0,06974
Root Mean Square Error	15,28353
Mean of Response	8,233769
Observations (or Sum Wgts)	23

Analysis of Variance

Source	DF	Sum of Squares	Mean Square	F Ratio
Model	3	365,7477	121,916	0,5219
Error	19	4438,1401	233,586	**Prob > F**
C. Total	22	4803,8878		0,6724

Parameter Estimates

Term	Estimate	Std Error	t Ratio	Prob>|t|
Intercept	8,3099928	3,508381	2,37	0,0286*
clasif[less60]	2,3412652	3,891163	0,60	0,5545
gender[female]	4,2647972	4,674934	0,91	0,3731
genetictype[black]	4,2294631	3,886667	1,09	0,2901

Effect Tests

Source	Nparm	DF	Sum of Squares	F Ratio	Prob > F
clasif	1	1	84,56483	0,3620	0,5545
gender	1	1	194,39864	0,8322	0,3731
genetictype	1	1	276,60702	1,1842	0,2901

clasif

Least Squares Means Table

Level	Least Sq Mean	Std Error	Mean
less60	10,651258	5,5377745	9,31999
S	5,968728	4,9226855	6,82169

gender

Least Squares Means Table

Level	Least Sq Mean	Std Error	Mean
female	12,574790	6,6298410	9,08983
male	4,045196	4,9368719	7,85924

genetictype

Least Squares Means Table

Level	Least Sq Mean	Std Error	Mean
black	12,539456	4,9474851	10,1924
white	4,080530	5,5092862	4,5614

Fit Group

Response Heptane

Whole Model

Effect Summary

Source	LogWorth	PValue
clasif	0,290	0,51268
genetictype	0,162	0,68839
gender	0,009	0,97986

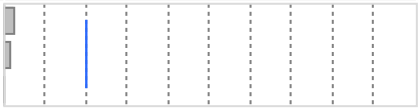

Lack Of Fit

Source	DF	Sum of Squares	Mean Square	F Ratio
Lack Of Fit	2	587,758	293,88	0,1558
Pure Error	17	32073,807	1886,69	**Prob > F**
Total Error	19	32661,565		0,8570
				**Max RSq**
				0,0691

Summary of Fit

RSquare	0,052059
RSquare Adj	-0,09762
Root Mean Square Error	41,46118
Mean of Response	32,21502
Observations (or Sum Wgts)	23

Analysis of Variance

Source	DF	Sum of Squares	Mean Square	F Ratio
Model	3	1793,710	597,90	0,3478
Error	19	32661,565	1719,03	**Prob > F**
C. Total	22	34455,275		0,7911

Parameter Estimates

Term	Estimate	Std Error	t Ratio	Prob>|t|
Intercept	30,116551	9,517542	3,16	0,0051*
clasif[less60]	7,042752	10,55595	0,67	0,5127
gender[female]	0,3243651	12,68217	0,03	0,9799
genetictype[black]	4,2936726	10,54376	0,41	0,6884

Effect Tests

Source	Nparm	DF	Sum of Squares	F Ratio	Prob > F
clasif	1	1	765,19718	0,4451	0,5127
gender	1	1	1,12451	0,0007	0,9799
genetictype	1	1	285,06937	0,1658	0,6884

clasif

Least Squares Means Table

Level	Least Sq Mean	Std Error	Mean
less60	37,159303	15,022882	39,1968
S	23,073799	13,354268	23,1387

gender

Least Squares Means Table

Level	Least Sq Mean	Std Error	Mean
female	30,440917	17,985441	23,5702
male	29,792186	13,392753	35,9971

genetictype

Least Squares Means Table

Level	Least Sq Mean	Std Error	Mean
black	34,410224	13,421544	36,5199
white	25,822879	14,945599	24,1433

Fit Group

Response Acetic acid

Whole Model

Effect Summary

Source	LogWorth	PValue
gender	0,465	0,34310
genetictype	0,259	0,55059
clasif	0,066	0,85937

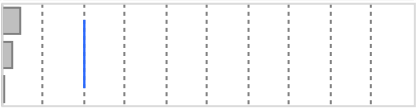

Lack Of Fit

Source	DF	Sum of Squares	Mean Square	F Ratio
Lack Of Fit	2	113,73051	56,8653	2,0329
Pure Error	17	475,53133	27,9724	**Prob > F**
Total Error	19	589,26185		0,1616
				**Max RSq**
				0,2368

Summary of Fit

RSquare	0,054248
RSquare Adj	-0,09508
Root Mean Square Error	5,569002
Mean of Response	4,162851
Observations (or Sum Wgts)	23

Analysis of Variance

Source	DF	Sum of Squares	Mean Square	F Ratio
Model	3	33,79966	11,2666	0,3633
Error	19	589,26185	31,0138	**Prob > F**
C. Total	22	623,06151		0,7802

Parameter Estimates

Term	Estimate	Std Error	t Ratio	Prob>|t|
Intercept	4,5158426	1,278381	3,53	0,0022*
clasif[less60]	0,2546499	1,417859	0,18	0,8594
gender[female]	1,6563362	1,703449	0,97	0,3431
genetictype[black]	0,8606105	1,416221	0,61	0,5506

Effect Tests

Source	Nparm	DF	Sum of Squares	F Ratio	Prob > F
clasif	1	1	1,000404	0,0323	0,8594
gender	1	1	29,321988	0,9455	0,3431
genetictype	1	1	11,452649	0,3693	0,5506

clasif

Least Squares Means Table

Level	Least Sq Mean	Std Error	Mean
less60	4,7704925	2,0178502	3,83238
S	4,2611927	1,7937245	4,59246

gender

Least Squares Means Table

Level	Least Sq Mean	Std Error	Mean
female	6,1721787	2,4157766	5,62145
male	2,8595064	1,7988938	3,52471

genetictype

Least Squares Means Table

Level	Least Sq Mean	Std Error	Mean
black	5,3764530	1,8027610	4,24669
white	3,6552321	2,0074697	4,00565

Fit Group

Response Furan, 2-ethyl-

Whole Model

Effect Summary

Source	LogWorth	PValue
genetictype	0,683	0,20757
gender	0,243	0,57135
clasif	0,015	0,96622

Lack Of Fit

Source	DF	Sum of Squares	Mean Square	F Ratio
Lack Of Fit	2	2,98853	1,4943	0,0359
Pure Error	17	706,71548	41,5715	**Prob > F**
Total Error	19	709,70401		0,9648
				**Max RSq**
				0,0862

Summary of Fit

RSquare	0,082294
RSquare Adj	-0,06261
Root Mean Square Error	6,111697
Mean of Response	8,904808
Observations (or Sum Wgts)	23

Analysis of Variance

Source	DF	Sum of Squares	Mean Square	F Ratio
Model	3	63,64149	21,2138	0,5679
Error	19	709,70401	37,3528	**Prob > F**
C. Total	22	773,34550		0,6429

Parameter Estimates

Term	Estimate	Std Error	t Ratio	Prob>|t|
Intercept	8,700311	1,402959	6,20	<,0001*
clasif[less60]	0,0667658	1,556029	0,04	0,9662
gender[female]	1,0768658	1,869449	0,58	0,5714
genetictype[black]	2,0278453	1,554231	1,30	0,2076

Effect Tests

Source	Nparm	DF	Sum of Squares	F Ratio	Prob > F
clasif	1	1	0,068770	0,0018	0,9662
gender	1	1	12,394232	0,3318	0,5714
genetictype	1	1	63,586124	1,7023	0,2076

clasif

Least Squares Means Table

Level	Least Sq Mean	Std Error	Mean
less60	8,7670768	2,2144883	8,94780
S	8,6335452	1,9685217	8,84892

gender

Least Squares Means Table

Level	Least Sq Mean	Std Error	Mean
female	9,7771768	2,6511924	8,86041
male	7,6234452	1,9741947	8,92423

genetictype

Least Squares Means Table

Level	Least Sq Mean	Std Error	Mean
black	10,728156	1,9784388	9,96071
white	6,672466	2,2030962	6,92499

Fit Group

Response 1-Butanol

Whole Model

Effect Summary

Source	LogWorth		PValue
clasif	1,743		0,01805
gender	1,580		0,02628
genetictype	1,221		0,06011

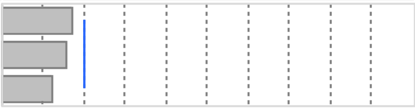

Lack Of Fit

Source	DF	Sum of Squares	Mean Square	F Ratio
Lack Of Fit	2	43,30988	21,6549	4,5678
Pure Error	17	80,59382	4,7408	**Prob > F**
Total Error	19	123,90370		0,0258*
				**Max RSq**
				0,5838

Summary of Fit

RSquare	0,36008
RSquare Adj	0,259041
Root Mean Square Error	2,553673
Mean of Response	4,684362
Observations (or Sum Wgts)	23

Analysis of Variance

Source	DF	Sum of Squares	Mean Square	F Ratio
Model	3	69,72018	23,2401	3,5637
Error	19	123,90370	6,5212	**Prob > F**
C. Total	22	193,62388		0,0337*

Parameter Estimates

Term	Estimate	Std Error	t Ratio	Prob>|t|
Intercept	4,8062925	0,586204	8,20	<,0001*
clasif[less60]	1,6824302	0,650161	2,59	0,0181*
gender[female]	1,8821502	0,781119	2,41	0,0263*
genetictype[black]	1,2982376	0,64941	2,00	0,0601

Effect Tests

Source	Nparm	DF	Sum of Squares	F Ratio	Prob > F
clasif	1	1	43,667939	6,6963	0,0181*
gender	1	1	37,862126	5,8060	0,0263*
genetictype	1	1	26,061599	3,9964	0,0601

clasif

Least Squares Means Table

Level	Least Sq Mean	Std Error	Mean
less60	6,4887227	0,92528794	5,59519
S	3,1238623	0,82251480	3,50029

gender

Least Squares Means Table

Level	Least Sq Mean	Std Error	Mean
female	6,6884427	1,1077576	4,93032
male	2,9241423	0,8248852	4,57676

genetictype

Least Squares Means Table

Level	Least Sq Mean	Std Error	Mean
black	6,1045301	0,82665848	5,28510
white	3,5080550	0,92052792	3,55798

Fit Group

Response 2-Pentanone

Whole Model

Effect Summary

Source	LogWorth	PValue
gender	0,865	0,13653
clasif	0,764	0,17205
genetictype	0,252	0,55942

Lack Of Fit

Source	DF	Sum of Squares	Mean Square	F Ratio
Lack Of Fit	2	40,08806	20,0440	1,0825
Pure Error	17	314,79340	18,5173	**Prob > F**
Total Error	19	354,88146		0,3610
				**Max RSq**
				0,2852

Summary of Fit

RSquare	0,194189
RSquare Adj	0,066955
Root Mean Square Error	4,321802
Mean of Response	3,939603
Observations (or Sum Wgts)	23

Analysis of Variance

Source	DF	Sum of Squares	Mean Square	F Ratio
Model	3	85,52122	28,5071	1,5262
Error	19	354,88146	18,6780	**Prob > F**
C. Total	22	440,40269		0,2401

Parameter Estimates

Term	Estimate	Std Error	t Ratio	Prob>|t|
Intercept	4,738862	0,992083	4,78	0,0001*
clasif[less60]	1,5615436	1,100324	1,42	0,1720
gender[female]	2,055174	1,321955	1,55	0,1365
genetictype[black]	-0,653002	1,099053	-0,59	0,5594

Effect Tests

Source	Nparm	DF	Sum of Squares	F Ratio	Prob > F
clasif	1	1	37,618094	2,0140	0,1720
gender	1	1	45,143335	2,4169	0,1365
genetictype	1	1	6,593589	0,3530	0,5594

clasif

Least Squares Means Table

Level	Least Sq Mean	Std Error	Mean
less60	6,3004056	1,5659447	4,20980
S	3,1773184	1,3920128	3,58835

gender

Least Squares Means Table

Level	Least Sq Mean	Std Error	Mean
female	6,7940360	1,8747539	5,95851
male	2,6836880	1,3960244	3,05633

genetictype

Least Squares Means Table

Level	Least Sq Mean	Std Error	Mean
black	4,0858599	1,3990255	3,09925
white	5,3918641	1,5578889	5,51527

Fit Group

Response Pentanal

Whole Model

Effect Summary

Source	LogWorth	PValue
clasif	0,833	0,14705
genetictype	0,728	0,18698
gender	0,476	0,33405

Lack Of Fit

Source	DF	Sum of Squares	Mean Square	F Ratio
Lack Of Fit	2	2200,15	1100,08	0,1299
Pure Error	17	143922,20	8466,01	**Prob > F**
Total Error	19	146122,35		0,8790
				**Max RSq**
				0,1994

Summary of Fit

RSquare	0,187137
RSquare Adj	0,05879
Root Mean Square Error	87,69635
Mean of Response	75,3971
Observations (or Sum Wgts)	23

Analysis of Variance

Source	DF	Sum of Squares	Mean Square	F Ratio
Model	3	33640,25	11213,4	1,4581
Error	19	146122,35	7690,7	**Prob > F**
C. Total	22	179762,61		0,2576

Parameter Estimates

Term	Estimate	Std Error	t Ratio	Prob>|t|
Intercept	78,687349	20,13097	3,91	0,0009*
clasif[less60]	-33,7536	22,32735	-1,51	0,1470
gender[female]	-26,58819	26,8246	-0,99	0,3341
genetictype[black]	-30,5298	22,30156	-1,37	0,1870

Effect Tests

Source	Nparm	DF	Sum of Squares	F Ratio	Prob > F
clasif	1	1	17576,351	2,2854	0,1470
gender	1	1	7555,687	0,9825	0,3341
genetictype	1	1	14412,542	1,8740	0,1870

clasif

Least Squares Means Table

Level	Least Sq Mean	Std Error	Mean
less60	44,93375	31,775551	50,992
S	112,44095	28,246193	107,123

gender

Least Squares Means Table

Level	Least Sq Mean	Std Error	Mean
female	52,09916	38,041789	89,2931
male	105,27554	28,327594	69,3176

genetictype

Least Squares Means Table

Level	Least Sq Mean	Std Error	Mean
black	48,15755	28,388492	56,404
white	109,21715	31,612086	111,008

Fit Group

Response 2,3-Pentanedione

Whole Model

Effect Summary

Source	LogWorth	PValue
clasif	0,588	0,25797
genetictype	0,218	0,60563
gender	0,184	0,65409

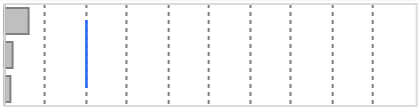

Lack Of Fit

Source	DF	Sum of Squares	Mean Square	F Ratio
Lack Of Fit	2	4,21069	2,10535	0,2271
Pure Error	17	157,60471	9,27087	**Prob > F**
Total Error	19	161,81540		0,7992
				**Max RSq**
				0,1131

Summary of Fit

RSquare	0,089426
RSquare Adj	-0,05435
Root Mean Square Error	2,918321
Mean of Response	3,275251
Observations (or Sum Wgts)	23

Analysis of Variance

Source	DF	Sum of Squares	Mean Square	F Ratio
Model	3	15,89163	5,29721	0,6220
Error	19	161,81540	8,51660	**Prob > F**
C. Total	22	177,70704		0,6094

Parameter Estimates

Term	Estimate	Std Error	t Ratio	Prob>|t|
Intercept	3,347841	0,66991	5,00	<,0001*
clasif[less60]	-0,866469	0,743	-1,17	0,2580
gender[female]	-0,406381	0,892658	-0,46	0,6541
genetictype[black]	-0,389657	0,742142	-0,53	0,6056

Effect Tests

Source	Nparm	DF	Sum of Squares	F Ratio	Prob > F
clasif	1	1	11,582300	1,3600	0,2580
gender	1	1	1,765072	0,2073	0,6541
genetictype	1	1	2,347777	0,2757	0,6056

clasif

Least Squares Means Table

Level	Least Sq Mean	Std Error	Mean
less60	2,4813718	1,0574131	2,61542
S	4,2143101	0,9399646	4,13303

gender

Least Squares Means Table

Level	Least Sq Mean	Std Error	Mean
female	2,9414604	1,2659383	3,72736
male	3,7542215	0,9426735	3,07745

genetictype

Least Squares Means Table

Level	Least Sq Mean	Std Error	Mean
black	2,9581843	0,9447000	2,96737
white	3,7374976	1,0519734	3,85252

Fit Group

Response Acetoin

Whole Model

Effect Summary

Source	LogWorth		PValue
clasif	0,366		0,43095
genetictype	0,110		0,77696
gender	0,040		0,91200

Lack Of Fit

Source	DF	Sum of Squares	Mean Square	F Ratio
Lack Of Fit	2	1904431	952216	0,3081
Pure Error	17	52533905	3090230	**Prob > F**
Total Error	19	54438336		0,7388
				**Max RSq**
				0,0840

Summary of Fit

RSquare	0,050742
RSquare Adj	-0,09914
Root Mean Square Error	1692,683
Mean of Response	1013,517
Observations (or Sum Wgts)	23

Analysis of Variance

Source	DF	Sum of Squares	Mean Square	F Ratio
Model	3	2909971	969990	0,3385
Error	19	54438336	2865176	**Prob > F**
C. Total	22	57348307		0,7977

Parameter Estimates

Term	Estimate	Std Error	t Ratio	Prob>|t|
Intercept	983,23706	388,5606	2,53	0,0204*
clasif[less60]	346,78593	430,9544	0,80	0,4310
gender[female]	-57,98811	517,7587	-0,11	0,9120
genetictype[black]	-123,6876	430,4565	-0,29	0,7770

Effect Tests

Source	Nparm	DF	Sum of Squares	F Ratio	Prob > F
clasif	1	1	1855288,7	0,6475	0,4310
gender	1	1	35939,7	0,0125	0,9120
genetictype	1	1	236562,0	0,0826	0,7770

clasif

Least Squares Means Table

Level	Least Sq Mean	Std Error	Mean
less60	1330,0230	613,32008	1312,49
S	636,4511	545,19769	624,85

gender

Least Squares Means Table

Level	Least Sq Mean	Std Error	Mean
female	925,2490	734,26872	730,55
male	1041,2252	546,76887	1137,31

genetictype

Least Squares Means Table

Level	Least Sq Mean	Std Error	Mean
black	859,5495	547,94430	1017,67
white	1106,9246	610,16494	1005,73

Fit Group

Response 3-Buten-1-ol, 3-methyl-

Whole Model

Effect Summary

Source	LogWorth	PValue
genetictype	0,376	0,42088
gender	0,319	0,47980
clasif	0,127	0,74561

Lack Of Fit

Source	DF	Sum of Squares	Mean Square	F Ratio
Lack Of Fit	2	750,863	375,431	0,5172
Pure Error	17	12339,250	725,838	**Prob > F**
Total Error	19	13090,113		0,6053
				**Max RSq**
				0,0977

Summary of Fit

RSquare	0,042771
RSquare Adj	-0,10837
Root Mean Square Error	26,24792
Mean of Response	17,38196
Observations (or Sum Wgts)	23

Analysis of Variance

Source	DF	Sum of Squares	Mean Square	F Ratio
Model	3	584,897	194,966	0,2830
Error	19	13090,113	688,953	**Prob > F**
C. Total	22	13675,010		0,8370

Parameter Estimates

Term	Estimate	Std Error	t Ratio	Prob>|t|
Intercept	17,68822	6,025291	2,94	0,0085*
clasif[less60]	2,1999438	6,682679	0,33	0,7456
gender[female]	5,7871845	8,028727	0,72	0,4798
genetictype[black]	5,4915566	6,674958	0,82	0,4209

Effect Tests

Source	Nparm	DF	Sum of Squares	F Ratio	Prob > F
clasif	1	1	74,66408	0,1084	0,7456
gender	1	1	357,95721	0,5196	0,4798
genetictype	1	1	466,31957	0,6769	0,4209

clasif

Least Squares Means Table

Level	Least Sq Mean	Std Error	Mean
less60	19,888163	9,5105682	17,9483
S	15,488276	8,4542149	16,6457

gender

Least Squares Means Table

Level	Least Sq Mean	Std Error	Mean
female	23,475404	11,386082	19,5505
male	11,901035	8,478579	16,4332

genetictype

Least Squares Means Table

Level	Least Sq Mean	Std Error	Mean
black	23,179776	8,4968057	19,6692
white	12,196663	9,4616424	13,0935

Fit Group

Response Toluene

Whole Model

Effect Summary

Source	LogWorth		PValue
clasif	0,185		0,65246
gender	0,048		0,89442
genetictype	0,006		0,98572

Lack Of Fit

Source	DF	Sum of Squares	Mean Square	F Ratio
Lack Of Fit	2	23,46334	11,7317	0,4115
Pure Error	17	484,68285	28,5108	**Prob > F**
Total Error	19	508,14618		0,6691
				**Max RSq**
				0,0576

Summary of Fit

RSquare	0,011936
RSquare Adj	-0,14407
Root Mean Square Error	5,171512
Mean of Response	9,245144
Observations (or Sum Wgts)	23

Analysis of Variance

Source	DF	Sum of Squares	Mean Square	F Ratio
Model	3	6,13824	2,0461	0,0765
Error	19	508,14618	26,7445	**Prob > F**
C. Total	22	514,28443		0,9719

Parameter Estimates

Term	Estimate	Std Error	t Ratio	Prob>|t|
Intercept	9,2477259	1,187136	7,79	<,0001*
clasif[less60]	-0,602443	1,316659	-0,46	0,6525
gender[female]	-0,212765	1,581865	-0,13	0,8944
genetictype[black]	-0,023848	1,315138	-0,02	0,9857

Effect Tests

Source	Nparm	DF	Sum of Squares	F Ratio	Prob > F
clasif	1	1	5,5991290	0,2094	0,6525
gender	1	1	0,4838362	0,0181	0,8944
genetictype	1	1	0,0087945	0,0003	0,9857

clasif

Least Squares Means Table

Level	Least Sq Mean	Std Error	Mean
less60	8,6452829	1,8738253	8,81247
S	9,8501689	1,6656967	9,80762

gender

Least Squares Means Table

Level	Least Sq Mean	Std Error	Mean
female	9,0349605	2,2433495	9,47550
male	9,4604913	1,6704970	9,14436

genetictype

Least Squares Means Table

Level	Least Sq Mean	Std Error	Mean
black	9,2238775	1,6740882	9,17909
white	9,2715744	1,8641857	9,36899

Fit Group

Response 1-Butanol, 3-methyl-

Whole Model

Effect Summary

Source	LogWorth	PValue
gender	0,477	0,33308
genetictype	0,379	0,41813
clasif	0,179	0,66297

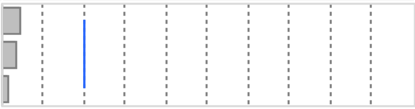

Lack Of Fit

Source	DF	Sum of Squares	Mean Square	F Ratio
Lack Of Fit	2	77939,6	38969,8	0,6660
Pure Error	17	994779,6	58516,4	**Prob > F**
Total Error	19	1072719,2		0,5267
				**Max RSq**
				0,1267

Summary of Fit

RSquare	0,058298
RSquare Adj	-0,09039
Root Mean Square Error	237,6108
Mean of Response	179,4704
Observations (or Sum Wgts)	23

Analysis of Variance

Source	DF	Sum of Squares	Mean Square	F Ratio
Model	3	66409,1	22136,4	0,3921
Error	19	1072719,2	56458,9	**Prob > F**
C. Total	22	1139128,3		0,7601

Parameter Estimates

Term	Estimate	Std Error	t Ratio	Prob>|t|
Intercept	189,00308	54,54429	3,47	0,0026*
clasif[less60]	26,782061	60,49534	0,44	0,6630
gender[female]	72,187807	72,68052	0,99	0,3331
genetictype[black]	50,013102	60,42545	0,83	0,4181

Effect Tests

Source	Nparm	DF	Sum of Squares	F Ratio	Prob > F
clasif	1	1	11065,641	0,1960	0,6630
gender	1	1	55696,018	0,9865	0,3331
genetictype	1	1	38677,670	0,6851	0,4181

clasif

Least Squares Means Table

Level	Least Sq Mean	Std Error	Mean
less60	215,78515	86,094972	181,633
S	162,22102	76,532273	176,659

gender

Least Squares Means Table

Level	Least Sq Mean	Std Error	Mean
female	261,19089	103,07317	220,627
male	116,81528	76,75283	161,464

genetictype

Least Squares Means Table

Level	Least Sq Mean	Std Error	Mean
black	239,01619	76,917828	195,006
white	138,98998	85,652069	150,341

Fit Group

Response 1-Octene

Whole Model

Effect Summary

Source	LogWorth	PValue
clasif	0,251	0,56088
genetictype	0,059	0,87304
gender	0,021	0,95219

Lack Of Fit

Source	DF	Sum of Squares	Mean Square	F Ratio
Lack Of Fit	2	470,858	235,43	0,1533
Pure Error	17	26105,565	1535,62	**Prob > F**
Total Error	19	26576,423		0,8590
				**Max RSq**
				0,0453

Summary of Fit

RSquare	0,028094
RSquare Adj	-0,12536
Root Mean Square Error	37,39999
Mean of Response	28,53244
Observations (or Sum Wgts)	23

Analysis of Variance

Source	DF	Sum of Squares	Mean Square	F Ratio
Model	3	768,232	256,08	0,1831
Error	19	26576,423	1398,76	**Prob > F**
C. Total	22	27344,655		0,9066

Parameter Estimates

Term	Estimate	Std Error	t Ratio	Prob>|t|
Intercept	27,600409	8,585282	3,21	0,0046*
clasif[less60]	5,6362894	9,521978	0,59	0,5609
gender[female]	0,6950705	11,43993	0,06	0,9522
genetictype[black]	1,5404938	9,510976	0,16	0,8730

Effect Tests

Source	Nparm	DF	Sum of Squares	F Ratio	Prob > F
clasif	1	1	490,08920	0,3504	0,5609
gender	1	1	5,16362	0,0037	0,9522
genetictype	1	1	36,69549	0,0262	0,8730

clasif

Least Squares Means Table

Level	Least Sq Mean	Std Error	Mean
less60	33,236699	13,551365	33,4781
S	21,964120	12,046194	22,1031

gender

Least Squares Means Table

Level	Least Sq Mean	Std Error	Mean
female	28,295480	16,223736	23,6093
male	26,905339	12,080909	30,6863

genetictype

Least Squares Means Table

Level	Least Sq Mean	Std Error	Mean
black	29,140903	12,106880	30,5099
white	26,059916	13,481651	24,8246

Fit Group

Response Cyclopropane, pentyl-

Whole Model

Effect Summary

Source	LogWorth	PValue
genetictype	0,718	0,19122
gender	0,197	0,63527
clasif	0,082	0,82874

Lack Of Fit

Source	DF	Sum of Squares	Mean Square	F Ratio
Lack Of Fit	2	1250,461	625,23	0,3812
Pure Error	17	27881,359	1640,08	**Prob > F**
Total Error	19	29131,820		0,6887
				**Max RSq**
				0,2244

Summary of Fit

RSquare	0,189589
RSquare Adj	0,061629
Root Mean Square Error	39,15678
Mean of Response	26,40251
Observations (or Sum Wgts)	23

Analysis of Variance

Source	DF	Sum of Squares	Mean Square	F Ratio
Model	3	6815,132	2271,71	1,4816
Error	19	29131,820	1533,25	**Prob > F**
C. Total	22	35946,953		0,2514

Parameter Estimates

Term	Estimate	Std Error	t Ratio	Prob>|t|
Intercept	19,750738	8,98856	2,20	0,0406*
clasif[less60]	2,1864149	9,969256	0,22	0,8287
gender[female]	-5,773698	11,9773	-0,48	0,6353
genetictype[black]	13,495455	9,957737	1,36	0,1912

Effect Tests

Source	Nparm	DF	Sum of Squares	F Ratio	Prob > F
clasif	1	1	73,7486	0,0481	0,8287
gender	1	1	356,2908	0,2324	0,6353
genetictype	1	1	2816,2277	1,8368	0,1912

clasif

Least Squares Means Table

Level	Least Sq Mean	Std Error	Mean
less60	21,937153	14,187915	34,0894
S	17,564323	12,612042	16,4096

gender

Least Squares Means Table

Level	Least Sq Mean	Std Error	Mean
female	13,977040	16,985816	6,6315
male	25,524436	12,648388	35,0523

genetictype

Least Squares Means Table

Level	Least Sq Mean	Std Error	Mean
black	33,246193	12,675579	38,2090
white	6,255283	14,114927	4,2653

Fit Group

Response 3-Hydroxy-3-methyl-2-butanone

Whole Model

Effect Summary

Source	LogWorth	PValue
genetictype	1,041	0,09092
clasif	0,176	0,66709
gender	0,165	0,68452

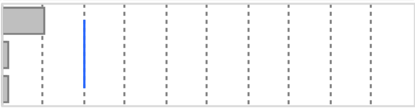

Lack Of Fit

Source	DF	Sum of Squares	Mean Square	F Ratio
Lack Of Fit	2	1,51994	0,7600	0,0585
Pure Error	17	220,83497	12,9903	**Prob > F**
Total Error	19	222,35492		0,9434
				**Max RSq**
				0,1582

Summary of Fit

RSquare	0,152408
RSquare Adj	0,018578
Root Mean Square Error	3,420949
Mean of Response	1,903344
Observations (or Sum Wgts)	23

Analysis of Variance

Source	DF	Sum of Squares	Mean Square	F Ratio
Model	3	39,98235	13,3275	1,1388
Error	19	222,35492	11,7029	**Prob > F**
C. Total	22	262,33727		0,3587

Parameter Estimates

Term	Estimate	Std Error	t Ratio	Prob>|t|
Intercept	2,1563027	0,785289	2,75	0,0128*
clasif[less60]	0,3805461	0,870968	0,44	0,6671
gender[female]	-0,431744	1,046401	-0,41	0,6845
genetictype[black]	-1,549341	0,869962	-1,78	0,0909

Effect Tests

Source	Nparm	DF	Sum of Squares	F Ratio	Prob > F
clasif	1	1	2,234103	0,1909	0,6671
gender	1	1	1,992274	0,1702	0,6845
genetictype	1	1	37,118187	3,1717	0,0909

clasif

Least Squares Means Table

Level	Least Sq Mean	Std Error	Mean
less60	2,5368488	1,2395331	2,06791
S	1,7757566	1,1018563	1,68941

gender

Least Squares Means Table

Level	Least Sq Mean	Std Error	Mean
female	1,7245587	1,4839729	2,11674
male	2,5880467	1,1050317	1,80998

genetictype

Least Squares Means Table

Level	Least Sq Mean	Std Error	Mean
black	0,6069617	1,1074072	1,05042
white	3,7056438	1,2331565	3,50257

Fit Group

Response Octane

Whole Model

Effect Summary

Source	LogWorth	PValue
clasif	0,230	0,58922
genetictype	0,120	0,75854
gender	0,010	0,97634

Lack Of Fit

Source	DF	Sum of Squares	Mean Square	F Ratio
Lack Of Fit	2	13803,82	6901,9	0,2231
Pure Error	17	525905,11	30935,6	**Prob > F**
Total Error	19	539708,93		0,8023
				**Max RSq**
				0,0628

Summary of Fit

RSquare	0,038239
RSquare Adj	-0,11362
Root Mean Square Error	168,54
Mean of Response	136,0709
Observations (or Sum Wgts)	23

Analysis of Variance

Source	DF	Sum of Squares	Mean Square	F Ratio
Model	3	21458,48	7152,8	0,2518
Error	19	539708,93	28405,7	**Prob > F**
C. Total	22	561167,40		0,8591

Parameter Estimates

Term	Estimate	Std Error	t Ratio	Prob>|t|
Intercept	128,32241	38,68887	3,32	0,0036*
clasif[less60]	23,569328	42,91002	0,55	0,5892
gender[female]	-1,549064	51,5531	-0,03	0,9763
genetictype[black]	13,366446	42,86044	0,31	0,7585

Effect Tests

Source	Nparm	DF	Sum of Squares	F Ratio	Prob > F
clasif	1	1	8570,0426	0,3017	0,5892
gender	1	1	25,6469	0,0009	0,9763
genetictype	1	1	2762,6421	0,0973	0,7585

clasif

Least Squares Means Table

Level	Least Sq Mean	Std Error	Mean
less60	151,89173	61,068122	160,400
S	104,75308	54,285193	104,443

gender

Least Squares Means Table

Level	Least Sq Mean	Std Error	Mean
female	126,77334	73,110947	104,210
male	129,87147	54,441635	150,010

genetictype

Least Squares Means Table

Level	Least Sq Mean	Std Error	Mean
black	141,68885	54,558672	150,681
white	114,95596	60,753966	108,676

Fit Group

Response 1-Pentanol

Whole Model

Effect Summary

Source	LogWorth	PValue
clasif	1,715	0,01929
genetictype	1,700	0,01995
gender	0,955	0,11092

Lack Of Fit

Source	DF	Sum of Squares	Mean Square	F Ratio
Lack Of Fit	2	19093,85	9546,92	1,1466
Pure Error	17	141548,49	8326,38	**Prob > F**
Total Error	19	160642,33		0,3411
				**Max RSq**
				0,4904

Summary of Fit

RSquare	0,42162
RSquare Adj	0,330297
Root Mean Square Error	91,95031
Mean of Response	304,1117
Observations (or Sum Wgts)	23

Analysis of Variance

Source	DF	Sum of Squares	Mean Square	F Ratio
Model	3	117102,88	39034,3	4,6168
Error	19	160642,33	8454,9	**Prob > F**
C. Total	22	277745,21		0,0137*

Parameter Estimates

Term	Estimate	Std Error	t Ratio	Prob>|t|
Intercept	296,62557	21,10748	14,05	<,0001*
clasif[less60]	59,851589	23,4104	2,56	0,0193*
gender[female]	47,0254	28,12581	1,67	0,1109
genetictype[black]	59,407819	23,38336	2,54	0,0200*

Effect Tests

Source	Nparm	DF	Sum of Squares	F Ratio	Prob > F
clasif	1	1	55263,697	6,5363	0,0193*
gender	1	1	23635,318	2,7955	0,1109
genetictype	1	1	54573,265	6,4547	0,0200*

clasif

Least Squares Means Table

Level	Least Sq Mean	Std Error	Mean
less60	356,47716	33,316914	348,675
S	236,77398	29,616354	246,179

gender

Least Squares Means Table

Level	Least Sq Mean	Std Error	Mean
female	343,65097	39,887114	275,439
male	249,60017	29,701704	316,656

genetictype

Least Squares Means Table

Level	Least Sq Mean	Std Error	Mean
black	356,03339	29,765556	341,499
white	237,21775	33,145520	234,011

Fit Group

Response Hexanal

Whole Model

Effect Summary

Source	LogWorth	PValue
genetictype	0,610	0,24544
clasif	0,544	0,28586
gender	0,400	0,39797

Lack Of Fit

Source	DF	Sum of Squares	Mean Square	F Ratio
Lack Of Fit	2	25311	12655	0,0064
Pure Error	17	33547393	1973376	**Prob > F**
Total Error	19	33572704		0,9936
				**Max RSq**
				0,1252

Summary of Fit

RSquare	0,124532
RSquare Adj	-0,0137
Root Mean Square Error	1329,28
Mean of Response	1215,99
Observations (or Sum Wgts)	23

Analysis of Variance

Source	DF	Sum of Squares	Mean Square	F Ratio
Model	3	4775572	1591857	0,9009
Error	19	33572704	1766984	**Prob > F**
C. Total	22	38348276		0,4590

Parameter Estimates

Term	Estimate	Std Error	t Ratio	Prob>|t|
Intercept	1250,192	305,1402	4,10	0,0006*
clasif[less60]	-371,6477	338,4325	-1,10	0,2859
gender[female]	-351,6024	406,6007	-0,86	0,3980
genetictype[black]	-405,1596	338,0415	-1,20	0,2454

Effect Tests

Source	Nparm	DF	Sum of Squares	F Ratio	Prob > F
clasif	1	1	2130843,4	1,2059	0,2859
gender	1	1	1321296,3	0,7478	0,3980
genetictype	1	1	2538312,4	1,4365	0,2454

clasif

Least Squares Means Table

Level	Least Sq Mean	Std Error	Mean
less60	878,5442	481,64596	957,89
S	1621,8397	428,14881	1551,52

gender

Least Squares Means Table

Level	Least Sq Mean	Std Error	Mean
female	898,5895	576,62805	1337,69
male	1601,7944	429,38267	1162,75

genetictype

Least Squares Means Table

Level	Least Sq Mean	Std Error	Mean
black	845,0323	430,30575	978,99
white	1655,3516	479,16820	1660,36

Fit Group

Response 2,3-Butanediol

Whole Model

Effect Summary

Source	LogWorth	PValue
genetictype	0,700	0,19966
clasif	0,556	0,27780
gender	0,293	0,50977

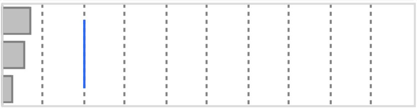

Lack Of Fit

Source	DF	Sum of Squares	Mean Square	F Ratio
Lack Of Fit	2	627,782	313,89	0,1317
Pure Error	11	26212,018	2382,91	**Prob > F**
Total Error	13	26839,800		0,8779
				**Max RSq**
				0,2341

Summary of Fit

RSquare	0,215739
RSquare Adj	0,034756
Root Mean Square Error	45,43787
Mean of Response	32,05374
Observations (or Sum Wgts)	17

Analysis of Variance

Source	DF	Sum of Squares	Mean Square	F Ratio
Model	3	7383,244	2461,08	1,1920
Error	13	26839,800	2064,60	**Prob > F**
C. Total	16	34223,044		0,3512

Parameter Estimates

Term	Estimate	Std Error	t Ratio	Prob>|t|
Intercept	43,147938	13,84729	3,12	0,0082*
clasif[less60]	14,717745	12,99362	1,13	0,2778
gender[female]	10,421107	15,37426	0,68	0,5098
genetictype[black]	-18,71113	13,84729	-1,35	0,1997

Effect Tests

Source	Nparm	DF	Sum of Squares	F Ratio	Prob > F
clasif	1	1	2648,8556	1,2830	0,2778
gender	1	1	948,5832	0,4595	0,5098
genetictype	1	1	3769,6985	1,8259	0,1997

clasif

Least Squares Means Table

Level	Least Sq Mean	Std Error	Mean
less60	57,865683	18,861537	37,4322
S	28,430193	19,115576	22,1932

gender

Least Squares Means Table

Level	Least Sq Mean	Std Error	Mean
female	53,569045	23,629611	46,2102
male	32,726831	17,258957	27,6979

genetictype

Least Squares Means Table

Level	Least Sq Mean	Std Error	Mean
black	24,436809	15,837906	22,8829
white	61,859067	22,718935	61,8591

Fit Group

Response 2,3-Butanediol, [S-(R*,R*)]-

Whole Model

Effect Summary

Source	LogWorth	PValue
clasif	0,473	0,33657
gender	0,145	0,71604
genetictype	0,027	0,93915

Lack Of Fit

Source	DF	Sum of Squares	Mean Square	F Ratio
Lack Of Fit	2	734,4998	367,250	0,9056
Pure Error	12	4866,1217	405,510	**Prob > F**
Total Error	14	5600,6215		0,4302
				**Max RSq**
				0,1903

Summary of Fit

RSquare	0,068099
RSquare Adj	-0,13159
Root Mean Square Error	20,00111
Mean of Response	18,28021
Observations (or Sum Wgts)	18

Analysis of Variance

Source	DF	Sum of Squares	Mean Square	F Ratio
Model	3	409,2675	136,422	0,3410
Error	14	5600,6215	400,044	**Prob > F**
C. Total	17	6009,8889		0,7961

Parameter Estimates

Term	Estimate	Std Error	t Ratio	Prob>|t|
Intercept	18,626662	5,880399	3,17	0,0068*
clasif[less60]	5,2675694	5,293486	1,00	0,3366
gender[female]	2,3894858	6,437026	0,37	0,7160
genetictype[black]	-0,426443	5,487045	-0,08	0,9392

Effect Tests

Source	Nparm	DF	Sum of Squares	F Ratio	Prob > F
clasif	1	1	396,13681	0,9902	0,3366
gender	1	1	55,12475	0,1378	0,7160
genetictype	1	1	2,41631	0,0060	0,9392

clasif

Least Squares Means Table

Level	Least Sq Mean	Std Error	Mean
less60	23,894232	7,9308377	21,7454
S	13,359093	7,8931613	12,8350

gender

Least Squares Means Table

Level	Least Sq Mean	Std Error	Mean
female	21,016148	10,344871	18,3824
male	16,237176	6,709132	18,2510

genetictype

Least Squares Means Table

Level	Least Sq Mean	Std Error	Mean
black	18,200220	6,9061789	17,7616
white	19,053105	9,0376000	19,6287

Fit Group

Response Butanoic acid

Whole Model

Effect Summary

Source	LogWorth	PValue
clasif	0,951	0,11194
gender	0,524	0,29942
genetictype	0,333	0,46476

Lack Of Fit

Source	DF	Sum of Squares	Mean Square	F Ratio
Lack Of Fit	2	12,061373	6,03069	4,9847
Pure Error	17	20,567177	1,20983	**Prob > F**
Total Error	19	32,628550		0,0198*
				**Max RSq**
				0,4637

Summary of Fit

RSquare	0,149182
RSquare Adj	0,014842
Root Mean Square Error	1,310455
Mean of Response	1,941381
Observations (or Sum Wgts)	23

Analysis of Variance

Source	DF	Sum of Squares	Mean Square	F Ratio
Model	3	5,721060	1,90702	1,1105
Error	19	32,628550	1,71729	**Prob > F**
C. Total	22	38,349611		0,3694

Parameter Estimates

Term	Estimate	Std Error	t Ratio	Prob>|t|
Intercept	1,9222571	0,300819	6,39	<,0001*
clasif[less60]	-0,556137	0,33364	-1,67	0,1119
gender[female]	-0,427628	0,400843	-1,07	0,2994
genetictype[black]	-0,248628	0,333254	-0,75	0,4648

Effect Tests

Source	Nparm	DF	Sum of Squares	F Ratio	Prob > F
clasif	1	1	4,7714760	2,7785	0,1119
gender	1	1	1,9544678	1,1381	0,2994
genetictype	1	1	0,9558566	0,5566	0,4648

clasif

Least Squares Means Table

Level	Least Sq Mean	Std Error	Mean
less60	1,3661197	0,47482509	1,59408
S	2,4783944	0,42208554	2,39287

gender

Least Squares Means Table

Level	Least Sq Mean	Std Error	Mean
female	1,4946291	0,56846208	1,99842
male	2,3498850	0,42330193	1,91642

genetictype

Least Squares Means Table

Level	Least Sq Mean	Std Error	Mean
black	1,6736291	0,42421193	1,80184
white	2,1708850	0,47238241	2,20301

Fit Group

Response Benzene, 1,3-dimethyl-

Whole Model

Effect Summary

Source	LogWorth	PValue
clasif	0,488	0,32499
genetictype	0,081	0,82961
gender	0,030	0,93424

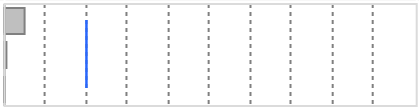

Lack Of Fit

Source	DF	Sum of Squares	Mean Square	F Ratio
Lack Of Fit	2	2,67321	1,3366	0,0465
Pure Error	17	488,26128	28,7213	**Prob > F**
Total Error	19	490,93450		0,9547
				**Max RSq**
				0,0810

Summary of Fit

RSquare	0,075975
RSquare Adj	-0,06992
Root Mean Square Error	5,083174
Mean of Response	5,074852
Observations (or Sum Wgts)	23

Analysis of Variance

Source	DF	Sum of Squares	Mean Square	F Ratio
Model	3	40,36527	13,4551	0,5207
Error	19	490,93450	25,8387	**Prob > F**
C. Total	22	531,29976		0,6731

Parameter Estimates

Term	Estimate	Std Error	t Ratio	Prob>|t|
Intercept	5,2803833	1,166858	4,53	0,0002*
clasif[less60]	-1,307643	1,294168	-1,01	0,3250
gender[female]	-0,13	1,554844	-0,08	0,9342
genetictype[black]	-0,282041	1,292673	-0,22	0,8296

Effect Tests

Source	Nparm	DF	Sum of Squares	F Ratio	Prob > F
clasif	1	1	26,379513	1,0209	0,3250
gender	1	1	0,180628	0,0070	0,9342
genetictype	1	1	1,230037	0,0476	0,8296

clasif

Least Squares Means Table

Level	Least Sq Mean	Std Error	Mean
less60	3,9727405	1,8418173	3,93087
S	6,5880260	1,6372438	6,56203

gender

Least Squares Means Table

Level	Least Sq Mean	Std Error	Mean
female	5,1503830	2,2050294	6,20529
male	5,4103835	1,6419621	4,58029

genetictype

Least Squares Means Table

Level	Least Sq Mean	Std Error	Mean
black	4,9983419	1,6454920	4,65779
white	5,5624246	1,8323423	5,85684

Fit Group

Response 1-Octen-3-one

Whole Model

Effect Summary

Source	LogWorth	PValue
genetictype	0,148	0,71074
clasif	0,109	0,77891
gender	0,105	0,78498

Lack Of Fit

Source	DF	Sum of Squares	Mean Square	F Ratio
Lack Of Fit	2	2,279062	1,13953	0,2986
Pure Error	17	64,880889	3,81652	**Prob > F**
Total Error	19	67,159951		0,7457
				**Max RSq**
				0,0532

Summary of Fit

RSquare	0,019928
RSquare Adj	-0,13482
Root Mean Square Error	1,880089
Mean of Response	2,347716
Observations (or Sum Wgts)	23

Analysis of Variance

Source	DF	Sum of Squares	Mean Square	F Ratio
Model	3	1,365579	0,45519	0,1288
Error	19	67,159951	3,53473	**Prob > F**
C. Total	22	68,525530		0,9418

Parameter Estimates

Term	Estimate	Std Error	t Ratio	Prob>|t|
Intercept	2,44699	0,43158	5,67	<,0001*
clasif[less60]	0,1363063	0,478668	0,28	0,7789
gender[female]	0,1591377	0,575083	0,28	0,7850
genetictype[black]	-0,179996	0,478115	-0,38	0,7107

Effect Tests

Source	Nparm	DF	Sum of Squares	F Ratio	Prob > F
clasif	1	1	0,28662904	0,0811	0,7789
gender	1	1	0,27067155	0,0766	0,7850
genetictype	1	1	0,50097779	0,1417	0,7107

clasif

Least Squares Means Table

Level	Least Sq Mean	Std Error	Mean
less60	2,5832963	0,68122402	2,35172
S	2,3106838	0,60555943	2,34251

gender

Least Squares Means Table

Level	Least Sq Mean	Std Error	Mean
female	2,6061278	0,81556352	2,58591
male	2,2878523	0,60730456	2,24351

genetictype

Least Squares Means Table

Level	Least Sq Mean	Std Error	Mean
black	2,2669940	0,60861013	2,19573
white	2,6269861	0,67771956	2,63269

Fit Group

Response Nonane

Whole Model

Effect Summary

Source	LogWorth	PValue
genetictype	0,886	0,13008
clasif	0,354	0,44268
gender	0,284	0,52057

Lack Of Fit

Source	DF	Sum of Squares	Mean Square	F Ratio
Lack Of Fit	2	5767,39	2883,7	0,2392
Pure Error	17	204959,87	12056,5	**Prob > F**
Total Error	19	210727,27		0,7899
				**Max RSq**
				0,1765

Summary of Fit

RSquare	0,153298
RSquare Adj	0,019608
Root Mean Square Error	105,3134
Mean of Response	123,538
Observations (or Sum Wgts)	23

Analysis of Variance

Source	DF	Sum of Squares	Mean Square	F Ratio
Model	3	38152,72	12717,6	1,1467
Error	19	210727,27	11090,9	**Prob > F**
C. Total	22	248879,99		0,3558

Parameter Estimates

Term	Estimate	Std Error	t Ratio	Prob>|t|
Intercept	116,14999	24,17501	4,80	0,0001*
clasif[less60]	21,022464	26,81262	0,78	0,4427
gender[female]	21,086809	32,21331	0,65	0,5206
genetictype[black]	42,376875	26,78164	1,58	0,1301

Effect Tests

Source	Nparm	DF	Sum of Squares	F Ratio	Prob > F
clasif	1	1	6817,981	0,6147	0,4427
gender	1	1	4752,457	0,4285	0,5206
genetictype	1	1	27768,382	2,5037	0,1301

clasif

Least Squares Means Table

Level	Least Sq Mean	Std Error	Mean
less60	137,17245	38,158837	142,148
S	95,12752	33,920477	99,345

gender

Least Squares Means Table

Level	Least Sq Mean	Std Error	Mean
female	137,23679	45,683879	104,059
male	95,06318	34,018231	132,060

genetictype

Least Squares Means Table

Level	Least Sq Mean	Std Error	Mean
black	158,52686	34,091362	150,071
white	73,77311	37,962534	73,789

Fit Group

Response 2-n-Butyl furan

Whole Model

Effect Summary

Source	LogWorth	PValue
genetictype	0,148	0,71085
gender	0,123	0,75386
clasif	0,017	0,96200

Lack Of Fit

Source	DF	Sum of Squares	Mean Square	F Ratio
Lack Of Fit	2	10,80961	5,40481	0,6743
Pure Error	17	136,26839	8,01579	**Prob > F**
Total Error	19	147,07800		0,5226
				**Max RSq**
				0,0832

Summary of Fit

RSquare	0,010512
RSquare Adj	-0,14572
Root Mean Square Error	2,782256
Mean of Response	4,243479
Observations (or Sum Wgts)	23

Analysis of Variance

Source	DF	Sum of Squares	Mean Square	F Ratio
Model	3	1,56245	0,52082	0,0673
Error	19	147,07800	7,74095	**Prob > F**
C. Total	22	148,64045		0,9766

Parameter Estimates

Term	Estimate	Std Error	t Ratio	Prob>|t|
Intercept	4,2728441	0,638675	6,69	<,0001*
clasif[less60]	-0,034196	0,708358	-0,05	0,9620
gender[female]	0,2707339	0,851038	0,32	0,7539
genetictype[black]	0,2662554	0,70754	0,38	0,7109

Effect Tests

Source	Nparm	DF	Sum of Squares	F Ratio	Prob > F
clasif	1	1	0,0180401	0,0023	0,9620
gender	1	1	0,7833968	0,1012	0,7539
genetictype	1	1	1,0961991	0,1416	0,7109

clasif

Least Squares Means Table

Level	Least Sq Mean	Std Error	Mean
less60	4,2386481	1,0081116	4,15293
S	4,3070401	0,8961391	4,36119

gender

Least Squares Means Table

Level	Least Sq Mean	Std Error	Mean
female	4,5435780	1,2069144	4,45389
male	4,0021102	0,8987217	4,15142

genetictype

Least Squares Means Table

Level	Least Sq Mean	Std Error	Mean
black	4,5390995	0,9006537	4,32916
white	4,0065888	1,0029255	4,08282

Fit Group

Response 1-Hexanol

Whole Model

Effect Summary

Source	LogWorth	PValue
genetictype	0,913	0,12221
clasif	0,382	0,41510
gender	0,289	0,51428

Lack Of Fit

Source	DF	Sum of Squares	Mean Square	F Ratio
Lack Of Fit	2	748512	374256	0,2352
Pure Error	17	27054762	1591457	**Prob > F**
Total Error	19	27803274		0,7930
				**Max RSq**
				0,1848

Summary of Fit

RSquare	0,162268
RSquare Adj	0,029994
Root Mean Square Error	1209,682
Mean of Response	1477,397
Observations (or Sum Wgts)	23

Analysis of Variance

Source	DF	Sum of Squares	Mean Square	F Ratio
Model	3	5385461	1795154	1,2268
Error	19	27803274	1463330	**Prob > F**
C. Total	22	33188735		0,3274

Parameter Estimates

Term	Estimate	Std Error	t Ratio	Prob>|t|
Intercept	1388,7008	277,6862	5,00	<,0001*
clasif[less60]	256,59967	307,9831	0,83	0,4151
gender[female]	245,92007	370,0181	0,66	0,5143
genetictype[black]	497,64104	307,6273	1,62	0,1222

Effect Tests

Source	Nparm	DF	Sum of Squares	F Ratio	Prob > F
clasif	1	1	1015782,6	0,6942	0,4151
gender	1	1	646374,8	0,4417	0,5143
genetictype	1	1	3829350,3	2,6169	0,1222

clasif

Least Squares Means Table

Level	Least Sq Mean	Std Error	Mean
less60	1645,3004	438,31137	1705,17
S	1132,1011	389,62746	1181,29

gender

Least Squares Means Table

Level	Least Sq Mean	Std Error	Mean
female	1634,6208	524,74774	1238,06
male	1142,7807	390,75031	1582,11

genetictype

Least Squares Means Table

Level	Least Sq Mean	Std Error	Mean
black	1886,3418	391,59033	1791,53
white	891,0597	436,05654	888,39

Fit Group

Response 2-Heptanone

Whole Model

Effect Summary

Source	LogWorth	PValue
clasif	0,468	0,34076
genetictype	0,258	0,55265
gender	0,170	0,67646

Lack Of Fit

Source	DF	Sum of Squares	Mean Square	F Ratio
Lack Of Fit	2	228,050	114,025	0,1939
Pure Error	17	9997,431	588,084	**Prob > F**
Total Error	19	10225,481		0,8255
				**Max RSq**
				0,0944

Summary of Fit

RSquare	0,073791
RSquare Adj	-0,07245
Root Mean Square Error	23,19878
Mean of Response	39,81581
Observations (or Sum Wgts)	23

Analysis of Variance

Source	DF	Sum of Squares	Mean Square	F Ratio
Model	3	814,667	271,556	0,5046
Error	19	10225,481	538,183	**Prob > F**
C. Total	22	11040,148		0,6837

Parameter Estimates

Term	Estimate	Std Error	t Ratio	Prob>|t|
Intercept	39,154385	5,32535	7,35	<,0001*
clasif[less60]	5,7715826	5,906372	0,98	0,3408
gender[female]	3,0074041	7,096053	0,42	0,6765
genetictype[black]	3,5663703	5,899548	0,60	0,5527

Effect Tests

Source	Nparm	DF	Sum of Squares	F Ratio	Prob > F
clasif	1	1	513,89974	0,9549	0,3408
gender	1	1	96,66739	0,1796	0,6765
genetictype	1	1	196,67338	0,3654	0,5527

clasif

Least Squares Means Table

Level	Least Sq Mean	Std Error	Mean
less60	44,925967	8,4057533	44,3016
S	33,382802	7,4721135	33,9843

gender

Least Squares Means Table

Level	Least Sq Mean	Std Error	Mean
female	42,161789	10,063394	36,5108
male	36,146981	7,493647	41,2618

genetictype

Least Squares Means Table

Level	Least Sq Mean	Std Error	Mean
black	42,720755	7,5097566	42,4392
white	35,588014	8,3625111	34,8970

Fit Group

Response Butanoic acid, 3-methyl-

Whole Model

Effect Summary

Source	LogWorth	PValue
clasif	0,437	0,36519
genetictype	0,030	0,93268
gender	0,026	0,94183

Lack Of Fit

Source	DF	Sum of Squares	Mean Square	F Ratio
Lack Of Fit	2	39,2438	19,622	0,1021
Pure Error	17	3268,5088	192,265	**Prob > F**
Total Error	19	3307,7526		0,9035
				**Max RSq**
				0,0660

Summary of Fit

RSquare	0,054834
RSquare Adj	-0,0944
Root Mean Square Error	13,1944
Mean of Response	5,536244
Observations (or Sum Wgts)	23

Analysis of Variance

Source	DF	Sum of Squares	Mean Square	F Ratio
Model	3	191,9003	63,967	0,3674
Error	19	3307,7526	174,092	**Prob > F**
C. Total	22	3499,6528		0,7773

Parameter Estimates

Term	Estimate	Std Error	t Ratio	Prob>|t|
Intercept	5,3339459	3,028815	1,76	0,0943
clasif[less60]	3,116437	3,359274	0,93	0,3652
gender[female]	0,2984424	4,03591	0,07	0,9418
genetictype[black]	-0,287212	3,355393	-0,09	0,9327

Effect Tests

Source	Nparm	DF	Sum of Squares	F Ratio	Prob > F
clasif	1	1	149,83224	0,8606	0,3652
gender	1	1	0,95196	0,0055	0,9418
genetictype	1	1	1,27555	0,0073	0,9327

clasif

Least Squares Means Table

Level	Least Sq Mean	Std Error	Mean
less60	8,4503829	4,7808077	8,04320
S	2,2175089	4,2497961	2,27720

gender

Least Squares Means Table

Level	Least Sq Mean	Std Error	Mean
female	5,6323883	5,7235979	3,52945
male	5,0355035	4,2620434	6,41421

genetictype

Least Squares Means Table

Level	Least Sq Mean	Std Error	Mean
black	5,0467343	4,2712058	5,86669
white	5,6211576	4,7562135	4,91666

Fit Group

Response Heptanal

Whole Model

Effect Summary

Source	LogWorth	PValue
clasif	0,592	0,25599
gender	0,427	0,37369
genetictype	0,002	0,99461

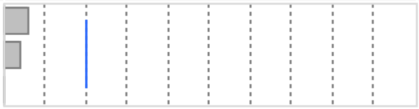

Lack Of Fit

Source	DF	Sum of Squares	Mean Square	F Ratio
Lack Of Fit	2	1483,334	741,67	0,3291
Pure Error	17	38316,998	2253,94	**Prob > F**
Total Error	19	39800,332		0,7241
				**Max RSq**
				0,1104

Summary of Fit

RSquare	0,07593
RSquare Adj	-0,06998
Root Mean Square Error	45,76849
Mean of Response	57,81559
Observations (or Sum Wgts)	23

Analysis of Variance

Source	DF	Sum of Squares	Mean Square	F Ratio
Model	3	3270,351	1090,12	0,5204
Error	19	39800,332	2094,75	**Prob > F**
C. Total	22	43070,683		0,6734

Parameter Estimates

Term	Estimate	Std Error	t Ratio	Prob>|t|
Intercept	54,580781	10,5063	5,20	<,0001*
clasif[less60]	-13,64784	11,65258	-1,17	0,2560
gender[female]	-12,75411	13,99969	-0,91	0,3737
genetictype[black]	0,0796022	11,63912	0,01	0,9946

Effect Tests

Source	Nparm	DF	Sum of Squares	F Ratio	Prob > F
clasif	1	1	2873,5325	1,3718	0,2560
gender	1	1	1738,5877	0,8300	0,3737
genetictype	1	1	0,0980	0,0000	0,9946

clasif

Least Squares Means Table

Level	Least Sq Mean	Std Error	Mean
less60	40,932946	16,583573	51,7677
S	68,228616	14,741611	65,6778

gender

Least Squares Means Table

Level	Least Sq Mean	Std Error	Mean
female	41,826673	19,853906	51,5410
male	67,334889	14,784094	60,5607

genetictype

Least Squares Means Table

Level	Least Sq Mean	Std Error	Mean
black	54,660383	14,815876	59,4641
white	54,501179	16,498261	54,7246

Fit Group

Response 2-Heptenal, (Z)-

Whole Model

Effect Summary

Source	LogWorth	PValue
genetictype	0,516	0,30514
clasif	0,084	0,82482
gender	0,036	0,92119

Lack Of Fit

Source	DF	Sum of Squares	Mean Square	F Ratio
Lack Of Fit	2	7,361430	3,68071	0,9963
Pure Error	17	62,805614	3,69445	**Prob > F**
Total Error	19	70,167043		0,3898
				**Max RSq**
				0,1717

Summary of Fit

RSquare	0,074617
RSquare Adj	-0,0715
Root Mean Square Error	1,921719
Mean of Response	5,067997
Observations (or Sum Wgts)	23

Analysis of Variance

Source	DF	Sum of Squares	Mean Square	F Ratio
Model	3	5,657814	1,88594	0,5107
Error	19	70,167043	3,69300	**Prob > F**
C. Total	22	75,824857		0,6797

Parameter Estimates

Term	Estimate	Std Error	t Ratio	Prob>|t|
Intercept	4,9025043	0,441136	11,11	<,0001*
clasif[less60]	-0,109804	0,489267	-0,22	0,8248
gender[female]	-0,058933	0,587816	-0,10	0,9212
genetictype[black]	0,5150502	0,488701	1,05	0,3051

Effect Tests

Source	Nparm	DF	Sum of Squares	F Ratio	Prob > F
clasif	1	1	0,1860048	0,0504	0,8248
gender	1	1	0,0371208	0,0101	0,9212
genetictype	1	1	4,1019639	1,1107	0,3051

clasif

Least Squares Means Table

Level	Least Sq Mean	Std Error	Mean
less60	4,7927004	0,69630795	5,11990
S	5,0123081	0,61896797	5,00052

gender

Least Squares Means Table

Level	Least Sq Mean	Std Error	Mean
female	4,8435710	0,83362205	4,70127
male	4,9614375	0,62075174	5,22844

genetictype

Least Squares Means Table

Level	Least Sq Mean	Std Error	Mean
black	5,4175545	0,62208621	5,42417
white	4,3874541	0,69272589	4,40017

Fit Group

Response Furan, 2-pentyl-

Whole Model

Effect Summary

Source	LogWorth	PValue
genetictype	0,343	0,45381
gender	0,076	0,84005
clasif	0,069	0,85394

Lack Of Fit

Source	DF	Sum of Squares	Mean Square	F Ratio
Lack Of Fit	2	5426,288	2713,14	0,6491
Pure Error	17	71059,012	4179,94	**Prob > F**
Total Error	19	76485,300		0,5350
				**Max RSq**
				0,1044

Summary of Fit

RSquare	0,035976
RSquare Adj	-0,11624
Root Mean Square Error	63,44716
Mean of Response	94,01814
Observations (or Sum Wgts)	23

Analysis of Variance

Source	DF	Sum of Squares	Mean Square	F Ratio
Model	3	2854,345	951,45	0,2364
Error	19	76485,300	4025,54	**Prob > F**
C. Total	22	79339,645		0,8699

Parameter Estimates

Term	Estimate	Std Error	t Ratio	Prob>|t|
Intercept	91,423367	14,56449	6,28	<,0001*
clasif[less60]	3,014408	16,15355	0,19	0,8539
gender[female]	3,9710467	19,40725	0,20	0,8400
genetictype[black]	12,339422	16,13488	0,76	0,4538

Effect Tests

Source	Nparm	DF	Sum of Squares	F Ratio	Prob > F
clasif	1	1	140,1821	0,0348	0,8539
gender	1	1	168,5413	0,0419	0,8400
genetictype	1	1	2354,4115	0,5849	0,4538

clasif

Least Squares Means Table

Level	Least Sq Mean	Std Error	Mean
less60	94,437775	22,989194	97,7220
S	88,408959	20,435750	89,2032

gender

Least Squares Means Table

Level	Least Sq Mean	Std Error	Mean
female	95,394414	27,522735	87,9529
male	87,452320	20,494642	96,6717

genetictype

Least Squares Means Table

Level	Least Sq Mean	Std Error	Mean
black	103,76279	20,538701	101,855
white	79,08394	22,870930	79,323

Fit Group

Response Benzaldehyde

Whole Model

Effect Summary

Source	LogWorth	PValue
genetictype	0,334	0,46387
gender	0,318	0,48098
clasif	0,059	0,87385

Lack Of Fit

Source	DF	Sum of Squares	Mean Square	F Ratio
Lack Of Fit	2	18,3218	9,1609	0,1467
Pure Error	17	1061,3182	62,4305	**Prob > F**
Total Error	19	1079,6400		0,8646
				**Max RSq**
				0,0547

Summary of Fit

RSquare	0,038357
RSquare Adj	-0,11348
Root Mean Square Error	7,538114
Mean of Response	8,977452
Observations (or Sum Wgts)	23

Analysis of Variance

Source	DF	Sum of Squares	Mean Square	F Ratio
Model	3	43,0633	14,3544	0,2526
Error	19	1079,6400	56,8232	**Prob > F**
C. Total	22	1122,7034		0,8585

Parameter Estimates

Term	Estimate	Std Error	t Ratio	Prob>|t|
Intercept	8,8053011	1,730397	5,09	<,0001*
clasif[less60]	-0,308858	1,919192	-0,16	0,8738
gender[female]	-1,657498	2,305762	-0,72	0,4810
genetictype[black]	-1,433064	1,916974	-0,75	0,4639

Effect Tests

Source	Nparm	DF	Sum of Squares	F Ratio	Prob > F
clasif	1	1	1,471652	0,0259	0,8738
gender	1	1	29,363137	0,5167	0,4810
genetictype	1	1	31,755853	0,5589	0,4639

clasif

Least Squares Means Table

Level	Least Sq Mean	Std Error	Mean
less60	8,4964435	2,7313305	9,12729
S	9,1141587	2,4279575	8,78266

gender

Least Squares Means Table

Level	Least Sq Mean	Std Error	Mean
female	7,147803	3,2699574	7,98259
male	10,462799	2,4349545	9,41271

genetictype

Least Squares Means Table

Level	Least Sq Mean	Std Error	Mean
black	7,372237	2,4401891	8,48478
white	10,238365	2,7172796	9,90120

Fit Group

Response 1-Heptanol

Whole Model

Effect Summary

Source	LogWorth	PValue
gender	0,352	0,44445
genetictype	0,030	0,93271
clasif	0,028	0,93716

Lack Of Fit

Source	DF	Sum of Squares	Mean Square	F Ratio
Lack Of Fit	2	1742,585	871,29	0,5418
Pure Error	17	27338,474	1608,15	**Prob > F**
Total Error	19	29081,059		0,5914
				**Max RSq**
				0,1121

Summary of Fit

RSquare	0,05553
RSquare Adj	-0,0936
Root Mean Square Error	39,12265
Mean of Response	39,58116
Observations (or Sum Wgts)	23

Analysis of Variance

Source	DF	Sum of Squares	Mean Square	F Ratio
Model	3	1709,815	569,94	0,3724
Error	19	29081,059	1530,58	**Prob > F**
C. Total	22	30790,874		0,7739

Parameter Estimates

Term	Estimate	Std Error	t Ratio	Prob>|t|
Intercept	43,393443	8,980725	4,83	0,0001*
clasif[less60]	0,795795	9,960566	0,08	0,9372
gender[female]	9,3456743	11,96686	0,78	0,4444
genetictype[black]	-0,85127	9,949058	-0,09	0,9327

Effect Tests

Source	Nparm	DF	Sum of Squares	F Ratio	Prob > F
clasif	1	1	9,76992	0,0064	0,9372
gender	1	1	933,50733	0,6099	0,4444
genetictype	1	1	11,20541	0,0073	0,9327

clasif

Least Squares Means Table

Level	Least Sq Mean	Std Error	Mean
less60	44,189238	14,175549	35,8230
S	42,597648	12,601049	44,4668

gender

Least Squares Means Table

Level	Least Sq Mean	Std Error	Mean
female	52,739117	16,971011	52,5355
male	34,047769	12,637363	33,9136

genetictype

Least Squares Means Table

Level	Least Sq Mean	Std Error	Mean
black	42,542172	12,664531	35,9539
white	44,244713	14,102624	46,3822

Fit Group

Response 2-Butenal, (Z)-

Whole Model

Effect Summary

Source	LogWorth	PValue
genetictype	0,736	0,18360
clasif	0,694	0,20217
gender	0,230	0,58840

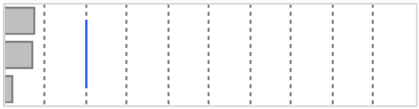

Lack Of Fit

Source	DF	Sum of Squares	Mean Square	F Ratio
Lack Of Fit	2	190,094	95,047	0,1158
Pure Error	17	13958,400	821,082	**Prob > F**
Total Error	19	14148,495		0,8914
				**Max RSq**
				0,2046

Summary of Fit

RSquare	0,193722
RSquare Adj	0,066415
Root Mean Square Error	27,28842
Mean of Response	39,74769
Observations (or Sum Wgts)	23

Analysis of Variance

Source	DF	Sum of Squares	Mean Square	F Ratio
Model	3	3399,412	1133,14	1,5217
Error	19	14148,495	744,66	**Prob > F**
C. Total	22	17547,907		0,2412

Parameter Estimates

Term	Estimate	Std Error	t Ratio	Prob>|t|
Intercept	37,433829	6,264139	5,98	<,0001*
clasif[less60]	9,1782569	6,947588	1,32	0,2022
gender[female]	4,5948511	8,346994	0,55	0,5884
genetictype[black]	9,5768057	6,93956	1,38	0,1836

Effect Tests

Source	Nparm	DF	Sum of Squares	F Ratio	Prob > F
clasif	1	1	1299,5979	1,7452	0,2022
gender	1	1	225,6521	0,3030	0,5884
genetictype	1	1	1418,1889	1,9045	0,1836

clasif

Least Squares Means Table

Level	Least Sq Mean	Std Error	Mean
less60	46,612086	9,8875771	47,8809
S	28,255572	8,7893489	29,1745

gender

Least Squares Means Table

Level	Least Sq Mean	Std Error	Mean
female	42,028680	11,837438	31,3684
male	32,838978	8,814678	43,4136

genetictype

Least Squares Means Table

Level	Least Sq Mean	Std Error	Mean
black	47,010635	8,8336280	46,7005
white	27,857023	9,8367119	26,7112

Fit Group

Response 1-Octen-3-ol

Whole Model

Effect Summary

Source	LogWorth	PValue
genetictype	0,654	0,22170
gender	0,312	0,48752
clasif	0,215	0,60998

Lack Of Fit

Source	DF	Sum of Squares	Mean Square	F Ratio
Lack Of Fit	2	18157,32	9078,7	0,3289
Pure Error	17	469312,62	27606,6	**Prob > F**
Total Error	19	487469,94		0,7242
				**Max RSq**
				0,1237

Summary of Fit

RSquare	0,089796
RSquare Adj	-0,05392
Root Mean Square Error	160,1759
Mean of Response	404,0656
Observations (or Sum Wgts)	23

Analysis of Variance

Source	DF	Sum of Squares	Mean Square	F Ratio
Model	3	48091,51	16030,5	0,6248
Error	19	487469,94	25656,3	**Prob > F**
C. Total	22	535561,45		0,6077

Parameter Estimates

Term	Estimate	Std Error	t Ratio	Prob>|t|
Intercept	399,21769	36,76886	10,86	<,0001*
clasif[less60]	21,15137	40,78053	0,52	0,6100
gender[female]	34,690155	48,99468	0,71	0,4875
genetictype[black]	51,465695	40,73341	1,26	0,2217

Effect Tests

Source	Nparm	DF	Sum of Squares	F Ratio	Prob > F
clasif	1	1	6901,851	0,2690	0,6100
gender	1	1	12862,012	0,5013	0,4875
genetictype	1	1	40957,024	1,5964	0,2217

clasif

Least Squares Means Table

Level	Least Sq Mean	Std Error	Mean
less60	420,36906	58,037498	418,728
S	378,06632	51,591185	385,004

gender

Least Squares Means Table

Level	Least Sq Mean	Std Error	Mean
female	433,90784	69,482674	396,743
male	364,52753	51,739863	407,269

genetictype

Least Squares Means Table

Level	Least Sq Mean	Std Error	Mean
black	450,68338	51,851092	432,294
white	347,75199	57,738932	351,137

Fit Group

Response 2-Octanone

Whole Model

Effect Summary

Source	LogWorth	PValue
clasif	0,660	0,21867
genetictype	0,066	0,85961
gender	0,006	0,98542

Lack Of Fit

Source	DF	Sum of Squares	Mean Square	F Ratio
Lack Of Fit	2	5,105252	2,55263	0,5157
Pure Error	17	84,153604	4,95021	**Prob > F**
Total Error	19	89,258856		0,6062
				**Max RSq**
				0,1580

Summary of Fit

RSquare	0,106895
RSquare Adj	-0,03412
Root Mean Square Error	2,167449
Mean of Response	2,71039
Observations (or Sum Wgts)	23

Analysis of Variance

Source	DF	Sum of Squares	Mean Square	F Ratio
Model	3	10,683263	3,56109	0,7580
Error	19	89,258856	4,69783	**Prob > F**
C. Total	22	99,942119		0,5314

Parameter Estimates

Term	Estimate	Std Error	t Ratio	Prob>|t|
Intercept	2,7766847	0,497545	5,58	<,0001*
clasif[less60]	-0,702015	0,551829	-1,27	0,2187
gender[female]	0,0122765	0,66298	0,02	0,9854
genetictype[black]	0,0988222	0,551191	0,18	0,8596

Effect Tests

Source	Nparm	DF	Sum of Squares	F Ratio	Prob > F
clasif	1	1	7,6029385	1,6184	0,2187
gender	1	1	0,0016108	0,0003	0,9854
genetictype	1	1	0,1510086	0,0321	0,8596

clasif

Least Squares Means Table

Level	Least Sq Mean	Std Error	Mean
less60	2,0746696	0,78534489	2,11749
S	3,4786998	0,69811544	3,48116

gender

Least Squares Means Table

Level	Least Sq Mean	Std Error	Mean
female	2,7889612	0,94021736	3,24805
male	2,7644081	0,70012730	2,47516

genetictype

Least Squares Means Table

Level	Least Sq Mean	Std Error	Mean
black	2,8755069	0,70163242	2,63250
white	2,6778625	0,78130479	2,85644

Fit Group

Response Acetic acid, butyl ester

Whole Model

Effect Summary

Source	LogWorth	PValue
clasif	0,774	0,16819
gender	0,443	0,36071
genetictype	0,046	0,89931

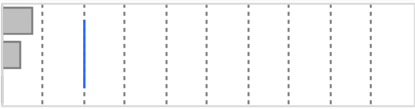

Lack Of Fit

Source	DF	Sum of Squares	Mean Square	F Ratio
Lack Of Fit	2	122,5673	61,284	0,4481
Pure Error	17	2325,1710	136,775	**Prob > F**
Total Error	19	2447,7383		0,6462
				**Max RSq**
				0,1488

Summary of Fit

RSquare	0,103956
RSquare Adj	-0,03752
Root Mean Square Error	11,35026
Mean of Response	14,33935
Observations (or Sum Wgts)	23

Analysis of Variance

Source	DF	Sum of Squares	Mean Square	F Ratio
Model	3	283,9797	94,660	0,7348
Error	19	2447,7383	128,828	**Prob > F**
C. Total	22	2731,7180		0,5441

Parameter Estimates

Term	Estimate	Std Error	t Ratio	Prob>|t|
Intercept	13,494303	2,605486	5,18	<,0001*
clasif[less60]	-4,140212	2,889758	-1,43	0,1682
gender[female]	-3,251756	3,471822	-0,94	0,3607
genetictype[black]	0,3701245	2,886419	0,13	0,8993

Effect Tests

Source	Nparm	DF	Sum of Squares	F Ratio	Prob > F
clasif	1	1	264,44402	2,0527	0,1682
gender	1	1	113,01404	0,8772	0,3607
genetictype	1	1	2,11830	0,0164	0,8993

clasif

Least Squares Means Table

Level	Least Sq Mean	Std Error	Mean
less60	9,354091	4,1126074	12,3049
S	17,634515	3,6558139	16,9842

gender

Least Squares Means Table

Level	Least Sq Mean	Std Error	Mean
female	10,242547	4,9236265	13,0412
male	16,746059	3,6663494	14,9073

genetictype

Least Squares Means Table

Level	Least Sq Mean	Std Error	Mean
black	13,864427	3,6742312	14,8690
white	13,124178	4,0914507	13,3463

Fit Group

Response Octanal

Whole Model

Effect Summary

Source	LogWorth	PValue
clasif	0,442	0,36179
gender	0,387	0,41033
genetictype	0,091	0,81032

Lack Of Fit

Source	DF	Sum of Squares	Mean Square	F Ratio
Lack Of Fit	2	170,7604	85,380	0,5351
Pure Error	17	2712,4569	159,556	**Prob > F**
Total Error	19	2883,2173		0,5952
				**Max RSq**
				0,1216

Summary of Fit

RSquare	0,066343
RSquare Adj	-0,08108
Root Mean Square Error	12,31862
Mean of Response	15,20331
Observations (or Sum Wgts)	23

Analysis of Variance

Source	DF	Sum of Squares	Mean Square	F Ratio
Model	3	204,8722	68,291	0,4500
Error	19	2883,2173	151,748	**Prob > F**
C. Total	22	3088,0895		0,7202

Parameter Estimates

Term	Estimate	Std Error	t Ratio	Prob>|t|
Intercept	14,112233	2,827776	4,99	<,0001*
clasif[less60]	-2,930759	3,1363	-0,93	0,3618
gender[female]	-3,172216	3,768024	-0,84	0,4103
genetictype[black]	0,7624473	3,132676	0,24	0,8103

Effect Tests

Source	Nparm	DF	Sum of Squares	F Ratio	Prob > F
clasif	1	1	132,51003	0,8732	0,3618
gender	1	1	107,55285	0,7088	0,4103
genetictype	1	1	8,98902	0,0592	0,8103

clasif

Least Squares Means Table

Level	Least Sq Mean	Std Error	Mean
less60	11,181474	4,4634786	14,2762
S	17,042992	3,9677132	16,4085

gender

Least Squares Means Table

Level	Least Sq Mean	Std Error	Mean
female	10,940017	5,3436905	12,7067
male	17,284449	3,9791476	16,2956

genetictype

Least Squares Means Table

Level	Least Sq Mean	Std Error	Mean
black	14,874681	3,9877018	16,2241
white	13,349786	4,4405168	13,2894

Fit Group

Response Hexane, 2,4,4-trimethyl-

Whole Model

Effect Summary

Source	LogWorth	PValue
clasif	1,803	0,01573
gender	0,091	0,81098
genetictype	0,041	0,90915

Lack Of Fit

Source	DF	Sum of Squares	Mean Square	F Ratio
Lack Of Fit	2	2338,42	1169,21	0,1632
Pure Error	17	121824,17	7166,13	**Prob > F**
Total Error	19	124162,58		0,8508
				**Max RSq**
				0,3446

Summary of Fit

RSquare	0,33203
RSquare Adj	0,226561
Root Mean Square Error	80,83856
Mean of Response	70,98631
Observations (or Sum Wgts)	23

Analysis of Variance

Source	DF	Sum of Squares	Mean Square	F Ratio
Model	3	61717,96	20572,7	3,1481
Error	19	124162,58	6534,9	**Prob > F**
C. Total	22	185880,55		0,0491*

Parameter Estimates

Term	Estimate	Std Error	t Ratio	Prob>|t|
Intercept	76,482682	18,55674	4,12	0,0006*
clasif[less60]	-54,58123	20,58137	-2,65	0,0157*
gender[female]	-5,996554	24,72694	-0,24	0,8110
genetictype[black]	-2,377396	20,55759	-0,12	0,9091

Effect Tests

Source	Nparm	DF	Sum of Squares	F Ratio	Prob > F
clasif	1	1	45959,483	7,0330	0,0157*
gender	1	1	384,326	0,0588	0,8110
genetictype	1	1	87,397	0,0134	0,9091

clasif

Least Squares Means Table

Level	Least Sq Mean	Std Error	Mean
less60	21,90146	29,290726	25,695
S	131,06391	26,037361	129,865

gender

Least Squares Means Table

Level	Least Sq Mean	Std Error	Mean
female	70,486128	35,066949	110,492
male	82,479236	26,112396	53,703

genetictype

Least Squares Means Table

Level	Least Sq Mean	Std Error	Mean
black	74,105286	26,168532	60,3090
white	78,860078	29,140044	91,0062

Fit Group

Response Pentanoic acid

Whole Model

Effect Summary

Source	LogWorth	PValue
clasif	0,330	0,46812
genetictype	0,103	0,78909
gender	0,051	0,89020

Lack Of Fit

Source	DF	Sum of Squares	Mean Square	F Ratio
Lack Of Fit	2	203,5730	101,787	1,8437
Pure Error	17	938,5334	55,208	**Prob > F**
Total Error	19	1142,1064		0,1885
				**Max RSq**
				0,2132

Summary of Fit

RSquare	0,042593
RSquare Adj	-0,10858
Root Mean Square Error	7,75312
Mean of Response	8,858002
Observations (or Sum Wgts)	23

Analysis of Variance

Source	DF	Sum of Squares	Mean Square	F Ratio
Model	3	50,8095	16,9365	0,2818
Error	19	1142,1064	60,1109	**Prob > F**
C. Total	22	1192,9159		0,8379

Parameter Estimates

Term	Estimate	Std Error	t Ratio	Prob>|t|
Intercept	9,0815837	1,779752	5,10	<,0001*
clasif[less60]	-1,461457	1,973932	-0,74	0,4681
gender[female]	-0,331814	2,371528	-0,14	0,8902
genetictype[black]	-0,534904	1,971651	-0,27	0,7891

Effect Tests

Source	Nparm	DF	Sum of Squares	F Ratio	Prob > F
clasif	1	1	32,950409	0,5482	0,4681
gender	1	1	1,176756	0,0196	0,8902
genetictype	1	1	4,424293	0,0736	0,7891

clasif

Least Squares Means Table

Level	Least Sq Mean	Std Error	Mean
less60	7,620126	2,8092349	7,6129
S	10,543041	2,4972089	10,4767

gender

Least Squares Means Table

Level	Least Sq Mean	Std Error	Mean
female	8,7497695	3,3632248	10,0229
male	9,4133979	2,5044055	8,3484

genetictype

Least Squares Means Table

Level	Least Sq Mean	Std Error	Mean
black	8,5466800	2,5097893	8,30286
white	9,6164874	2,7947831	9,89890

Fit Group

Response Heptane, 3,3,4-trimethyl-

Whole Model

Effect Summary

Source	LogWorth	PValue
genetictype	0,144	0,71788
gender	0,090	0,81193
clasif	0,042	0,90715

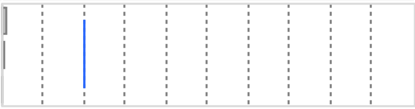

Lack Of Fit

Source	DF	Sum of Squares	Mean Square	F Ratio
Lack Of Fit	2	4,014478	2,00724	0,6728
Pure Error	17	50,715239	2,98325	**Prob > F**
Total Error	19	54,729717		0,5233
				**Max RSq**
				0,0988

Summary of Fit

RSquare	0,027437
RSquare Adj	-0,12613
Root Mean Square Error	1,697207
Mean of Response	3,28477
Observations (or Sum Wgts)	23

Analysis of Variance

Source	DF	Sum of Squares	Mean Square	F Ratio
Model	3	1,544000	0,51467	0,1787
Error	19	54,729717	2,88051	**Prob > F**
C. Total	22	56,273716		0,9095

Parameter Estimates

Term	Estimate	Std Error	t Ratio	Prob>|t|
Intercept	3,1809221	0,389599	8,16	<,0001*
clasif[less60]	0,051077	0,432106	0,12	0,9071
gender[female]	-0,125257	0,519142	-0,24	0,8119
genetictype[black]	0,1582784	0,431607	0,37	0,7179

Effect Tests

Source	Nparm	DF	Sum of Squares	F Ratio	Prob > F
clasif	1	1	0,04024761	0,0140	0,9071
gender	1	1	0,16768689	0,0582	0,8119
genetictype	1	1	0,38737873	0,1345	0,7179

clasif

Least Squares Means Table

Level	Least Sq Mean	Std Error	Mean
less60	3,2319991	0,61495929	3,42321
S	3,1298450	0,54665482	3,10479

gender

Least Squares Means Table

Level	Least Sq Mean	Std Error	Mean
female	3,0556653	0,73623118	2,95135
male	3,3061789	0,54823020	3,43064

genetictype

Least Squares Means Table

Level	Least Sq Mean	Std Error	Mean
black	3,3392004	0,54940877	3,44808
white	3,0226437	0,61179571	2,97856

Fit Group

Response 2-Ethyl-1-hexanol

Whole Model

Effect Summary

Source	LogWorth	PValue
gender	0,172	0,67282
genetictype	0,111	0,77374
clasif	0,033	0,92756

Lack Of Fit

Source	DF	Sum of Squares	Mean Square	F Ratio
Lack Of Fit	2	6,993382	3,49669	1,0521
Pure Error	17	56,502335	3,32367	**Prob > F**
Total Error	19	63,495717		0,3709
				**Max RSq**
				0,1246

Summary of Fit

RSquare	0,016278
RSquare Adj	-0,13905
Root Mean Square Error	1,828081
Mean of Response	3,772581
Observations (or Sum Wgts)	23

Analysis of Variance

Source	DF	Sum of Squares	Mean Square	F Ratio
Model	3	1,050704	0,35023	0,1048
Error	19	63,495717	3,34188	**Prob > F**
C. Total	22	64,546421		0,9563

Parameter Estimates

Term	Estimate	Std Error	t Ratio	Prob>|t|
Intercept	3,8307596	0,419642	9,13	<,0001*
clasif[less60]	-0,042879	0,465427	-0,09	0,9276
gender[female]	0,239828	0,559174	0,43	0,6728
genetictype[black]	0,1355704	0,464889	0,29	0,7737

Effect Tests

Source	Nparm	DF	Sum of Squares	F Ratio	Prob > F
clasif	1	1	0,02836488	0,0085	0,9276
gender	1	1	0,61474685	0,1840	0,6728
genetictype	1	1	0,28419914	0,0850	0,7737

clasif

Least Squares Means Table

Level	Least Sq Mean	Std Error	Mean
less60	3,7878804	0,66237965	3,65795
S	3,8736387	0,58880814	3,92160

gender

Least Squares Means Table

Level	Least Sq Mean	Std Error	Mean
female	4,0705876	0,79300299	4,04311
male	3,5909315	0,59050499	3,65422

genetictype

Least Squares Means Table

Level	Least Sq Mean	Std Error	Mean
black	3,9663300	0,59177444	3,77616
white	3,6951891	0,65897213	3,76587

Fit Group

Response Undecane

Whole Model

Effect Summary

Source	LogWorth	PValue
clasif	0,207	0,62073
gender	0,069	0,85266
genetictype	0,013	0,96977

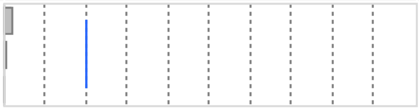

Lack Of Fit

Source	DF	Sum of Squares	Mean Square	F Ratio
Lack Of Fit	2	1589,875	794,938	1,3099
Pure Error	17	10316,538	606,855	**Prob > F**
Total Error	19	11906,414		0,2957
				**Max RSq**
				0,1452

Summary of Fit

RSquare	0,013462
RSquare Adj	-0,14231
Root Mean Square Error	25,03305
Mean of Response	59,81446
Observations (or Sum Wgts)	23

Analysis of Variance

Source	DF	Sum of Squares	Mean Square	F Ratio
Model	3	162,474	54,158	0,0864
Error	19	11906,414	626,653	**Prob > F**
C. Total	22	12068,888		0,9666

Parameter Estimates

Term	Estimate	Std Error	t Ratio	Prob>|t|
Intercept	59,594105	5,746412	10,37	<,0001*
clasif[less60]	-3,205905	6,373374	-0,50	0,6207
gender[female]	-1,441638	7,65712	-0,19	0,8527
genetictype[black]	0,2444487	6,36601	0,04	0,9698

Effect Tests

Source	Nparm	DF	Sum of Squares	F Ratio	Prob > F
clasif	1	1	158,55864	0,2530	0,6207
gender	1	1	22,21307	0,0354	0,8527
genetictype	1	1	0,92399	0,0015	0,9698

clasif

Least Squares Means Table

Level	Least Sq Mean	Std Error	Mean
less60	56,388200	9,0703751	57,7397
S	62,800010	8,0629147	62,5117

gender

Least Squares Means Table

Level	Least Sq Mean	Std Error	Mean
female	58,152468	10,859081	60,3376
male	61,035743	8,086151	59,5856

genetictype

Least Squares Means Table

Level	Least Sq Mean	Std Error	Mean
black	59,838554	8,1035342	59,8271
white	59,349657	9,0237138	59,7907

Fit Group

Response 2-Octenal, (E)-

Whole Model

Effect Summary

Source	LogWorth	PValue
clasif	1,016	0,09632
gender	0,379	0,41785
genetictype	0,324	0,47449

Lack Of Fit

Source	DF	Sum of Squares	Mean Square	F Ratio
Lack Of Fit	2	4,57174	2,2859	0,0565
Pure Error	17	687,56978	40,4453	**Prob > F**
Total Error	19	692,14151		0,9452
				**Max RSq**
				0,1732

Summary of Fit

RSquare	0,167662
RSquare Adj	0,03624
Root Mean Square Error	6,035603
Mean of Response	4,902771
Observations (or Sum Wgts)	23

Analysis of Variance

Source	DF	Sum of Squares	Mean Square	F Ratio
Model	3	139,42142	46,4738	1,2758
Error	19	692,14151	36,4285	**Prob > F**
C. Total	22	831,56293		0,3111

Parameter Estimates

Term	Estimate	Std Error	t Ratio	Prob>|t|
Intercept	4,8095235	1,385491	3,47	0,0026*
clasif[less60]	2,6885324	1,536655	1,75	0,0963
gender[female]	1,5289628	1,846173	0,83	0,4179
genetictype[black]	1,1199645	1,53488	0,73	0,4745

Effect Tests

Source	Nparm	DF	Sum of Squares	F Ratio	Prob > F
clasif	1	1	111,51136	3,0611	0,0963
gender	1	1	24,98563	0,6859	0,4179
genetictype	1	1	19,39551	0,5324	0,4745

clasif

Least Squares Means Table

Level	Least Sq Mean	Std Error	Mean
less60	7,4980560	2,1869166	6,80738
S	2,1209911	1,9440124	2,42678

gender

Least Squares Means Table

Level	Least Sq Mean	Std Error	Mean
female	6,3384863	2,6181833	3,93812
male	3,2805608	1,9496148	5,32480

genetictype

Least Squares Means Table

Level	Least Sq Mean	Std Error	Mean
black	5,9294881	1,9538060	5,70443
white	3,6895590	2,1756663	3,39967

Fit Group

Response 1-Octanol

Whole Model

Effect Summary

Source	LogWorth	PValue
genetictype	0,581	0,26217
clasif	0,312	0,48777
gender	0,019	0,95787

Lack Of Fit

Source	DF	Sum of Squares	Mean Square	F Ratio
Lack Of Fit	2	140,1332	70,067	0,4040
Pure Error	17	2948,1562	173,421	**Prob > F**
Total Error	19	3088,2894		0,6739
				**Max RSq**
				0,1886

Summary of Fit

RSquare	0,150057
RSquare Adj	0,015856
Root Mean Square Error	12,74918
Mean of Response	18,88844
Observations (or Sum Wgts)	23

Analysis of Variance

Source	DF	Sum of Squares	Mean Square	F Ratio
Model	3	545,2379	181,746	1,1182
Error	19	3088,2894	162,542	**Prob > F**
C. Total	22	3633,5273		0,3665

Parameter Estimates

Term	Estimate	Std Error	t Ratio	Prob>|t|
Intercept	17,36687	2,926613	5,93	<,0001*
clasif[less60]	2,2969034	3,245921	0,71	0,4878
gender[female]	-0,208738	3,899725	-0,05	0,9579
genetictype[black]	3,7466629	3,24217	1,16	0,2622

Effect Tests

Source	Nparm	DF	Sum of Squares	F Ratio	Prob > F
clasif	1	1	81,39056	0,5007	0,4878
gender	1	1	0,46569	0,0029	0,9579
genetictype	1	1	217,06108	1,3354	0,2622

clasif

Least Squares Means Table

Level	Least Sq Mean	Std Error	Mean
less60	19,663774	4,6194871	21,8578
S	15,069967	4,1063937	15,0282

gender

Least Squares Means Table

Level	Least Sq Mean	Std Error	Mean
female	17,158132	5,5304644	13,9118
male	17,575609	4,1182277	21,0657

genetictype

Least Squares Means Table

Level	Least Sq Mean	Std Error	Mean
black	21,113533	4,1270809	22,0322
white	13,620207	4,5957228	12,9938

Fit Group

Response Heptane, 4-methylene-

Whole Model

Effect Summary

Source	LogWorth	PValue
genetictype	0,677	0,21035
clasif	0,475	0,33489
gender	0,064	0,86286

Lack Of Fit

Source	DF	Sum of Squares	Mean Square	F Ratio
Lack Of Fit	2	178,7355	89,368	0,5953
Pure Error	17	2552,0570	150,121	**Prob > F**
Total Error	19	2730,7925		0,5625
				**Max RSq**
				0,2309

Summary of Fit

RSquare	0,177084
RSquare Adj	0,047149
Root Mean Square Error	11,98857
Mean of Response	16,33721
Observations (or Sum Wgts)	23

Analysis of Variance

Source	DF	Sum of Squares	Mean Square	F Ratio
Model	3	587,6398	195,880	1,3629
Error	19	2730,7925	143,726	**Prob > F**
C. Total	22	3318,4323		0,2842

Parameter Estimates

Term	Estimate	Std Error	t Ratio	Prob>|t|
Intercept	14,991597	2,752014	5,45	<,0001*
clasif[less60]	3,0200075	3,052272	0,99	0,3349
gender[female]	0,6420858	3,667071	0,18	0,8629
genetictype[black]	3,9525451	3,048746	1,30	0,2103

Effect Tests

Source	Nparm	DF	Sum of Squares	F Ratio	Prob > F
clasif	1	1	140,70341	0,9790	0,3349
gender	1	1	4,40639	0,0307	0,8629
genetictype	1	1	241,57188	1,6808	0,2103

clasif

Least Squares Means Table

Level	Least Sq Mean	Std Error	Mean
less60	18,011604	4,3438929	19,5966
S	11,971589	3,8614102	12,1000

gender

Least Squares Means Table

Level	Least Sq Mean	Std Error	Mean
female	15,633683	5,2005222	11,7826
male	14,349511	3,8725382	18,3299

genetictype

Least Squares Means Table

Level	Least Sq Mean	Std Error	Mean
black	18,944142	3,8808632	19,4799
white	11,039052	4,3215464	10,4446

Fit Group

Response Pentanoic acid, 2-methyl-, anhydride

Whole Model

Effect Summary

Source	LogWorth	PValue
genetictype	0,153	0,70265
clasif	0,045	0,90246
gender	0,007	0,98487

Lack Of Fit

Source	DF	Sum of Squares	Mean Square	F Ratio
Lack Of Fit	2	213,8912	106,946	1,4733
Pure Error	17	1234,0057	72,589	**Prob > F**
Total Error	19	1447,8969		0,2570
				**Max RSq**
				0,1557

Summary of Fit

RSquare	0,009414
RSquare Adj	-0,14699
Root Mean Square Error	8,729553
Mean of Response	14,70287
Observations (or Sum Wgts)	23

Analysis of Variance

Source	DF	Sum of Squares	Mean Square	F Ratio
Model	3	13,7602	4,5867	0,0602
Error	19	1447,8969	76,2051	**Prob > F**
C. Total	22	1461,6570		0,9801

Parameter Estimates

Term	Estimate	Std Error	t Ratio	Prob>|t|
Intercept	14,497107	2,003896	7,23	<,0001*
clasif[less60]	-0,276053	2,222531	-0,12	0,9025
gender[female]	0,0513088	2,6702	0,02	0,9849
genetictype[black]	0,8603659	2,219963	0,39	0,7027

Effect Tests

Source	Nparm	DF	Sum of Squares	F Ratio	Prob > F
clasif	1	1	1,175642	0,0154	0,9025
gender	1	1	0,028137	0,0004	0,9849
genetictype	1	1	11,446142	0,1502	0,7027

clasif

Least Squares Means Table

Level	Least Sq Mean	Std Error	Mean
less60	14,221054	3,1630320	14,6409
S	14,773161	2,8117092	14,7834

gender

Least Squares Means Table

Level	Least Sq Mean	Std Error	Mean
female	14,548416	3,7867918	14,3769
male	14,445799	2,8198121	14,8455

genetictype

Least Squares Means Table

Level	Least Sq Mean	Std Error	Mean
black	15,357473	2,8258741	15,2278
white	13,636742	3,1467602	13,7186

Fit Group

Response Nonanal

Whole Model

Effect Summary

Source	LogWorth	PValue
clasif	0,306	0,49446
gender	0,215	0,60929
genetictype	0,019	0,95706

Lack Of Fit

Source	DF	Sum of Squares	Mean Square	F Ratio
Lack Of Fit	2	268,5006	134,250	0,8497
Pure Error	17	2685,8904	157,994	**Prob > F**
Total Error	19	2954,3910		0,4449
				**Max RSq**
				0,1167

Summary of Fit

RSquare	0,028425
RSquare Adj	-0,12498
Root Mean Square Error	12,46973
Mean of Response	15,761
Observations (or Sum Wgts)	23

Analysis of Variance

Source	DF	Sum of Squares	Mean Square	F Ratio
Model	3	86,4370	28,812	0,1853
Error	19	2954,3910	155,494	**Prob > F**
C. Total	22	3040,8280		0,9051

Parameter Estimates

Term	Estimate	Std Error	t Ratio	Prob>|t|
Intercept	15,221201	2,862466	5,32	<,0001*
clasif[less60]	-2,21171	3,174775	-0,70	0,4945
gender[female]	-1,982173	3,814248	-0,52	0,6093
genetictype[black]	0,1729913	3,171106	0,05	0,9571

Effect Tests

Source	Nparm	DF	Sum of Squares	F Ratio	Prob > F
clasif	1	1	75,464875	0,4853	0,4945
gender	1	1	41,993259	0,2701	0,6093
genetictype	1	1	0,462745	0,0030	0,9571

clasif

Least Squares Means Table

Level	Least Sq Mean	Std Error	Mean
less60	13,009491	4,5182343	14,7799
S	17,432911	4,0163872	17,0365

gender

Least Squares Means Table

Level	Least Sq Mean	Std Error	Mean
female	13,239028	5,4092442	14,7447
male	17,203374	4,0279618	16,2056

genetictype

Least Squares Means Table

Level	Least Sq Mean	Std Error	Mean
black	15,394192	4,0366210	16,1105
white	15,048210	4,4949909	15,1056

Fit Group

Response Phenylethyl Alcohol

Whole Model

Effect Summary

Source	LogWorth	PValue
gender	1,380	0,04169
clasif	0,745	0,17999
genetictype	0,341	0,45598

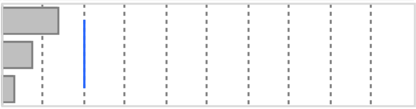

Lack Of Fit

Source	DF	Sum of Squares	Mean Square	F Ratio
Lack Of Fit	2	312,1901	156,095	2,4644
Pure Error	17	1076,7992	63,341	**Prob > F**
Total Error	19	1388,9893		0,1149
				**Max RSq**
				0,3836

Summary of Fit

RSquare	0,204919
RSquare Adj	0,07938
Root Mean Square Error	8,550129
Mean of Response	6,35251
Observations (or Sum Wgts)	23

Analysis of Variance

Source	DF	Sum of Squares	Mean Square	F Ratio
Model	3	357,9892	119,330	1,6323
Error	19	1388,9893	73,105	**Prob > F**
C. Total	22	1746,9785		0,2153

Parameter Estimates

Term	Estimate	Std Error	t Ratio	Prob>|t|
Intercept	7,6889092	1,962708	3,92	0,0009*
clasif[less60]	3,0302625	2,176849	1,39	0,1800
gender[female]	5,7123617	2,615318	2,18	0,0417*
genetictype[black]	1,6547555	2,174334	0,76	0,4560

Effect Tests

Source	Nparm	DF	Sum of Squares	F Ratio	Prob > F
clasif	1	1	141,66059	1,9378	0,1800
gender	1	1	348,76094	4,7707	0,0417*
genetictype	1	1	42,34093	0,5792	0,4560

clasif

Least Squares Means Table

Level	Least Sq Mean	Std Error	Mean
less60	10,719172	3,0980199	6,77666
S	4,658647	2,7539182	5,80112

gender

Least Squares Means Table

Level	Least Sq Mean	Std Error	Mean
female	13,401271	3,7089592	10,5276
male	1,976548	2,7618545	4,5259

genetictype

Least Squares Means Table

Level	Least Sq Mean	Std Error	Mean
black	9,3436648	2,7677919	6,16469
white	6,0341537	3,0820826	6,70468

Fit Group

Response Dodecane

Whole Model

Effect Summary

Source	LogWorth	PValue
clasif	0,243	0,57106
gender	0,153	0,70268
genetictype	0,013	0,97029

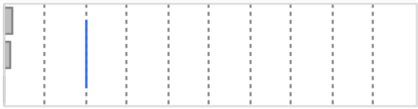

Lack Of Fit

Source	DF	Sum of Squares	Mean Square	F Ratio
Lack Of Fit	2	739,5177	369,759	1,6467
Pure Error	17	3817,3650	224,551	**Prob > F**
Total Error	19	4556,8826		0,2220
				**Max RSq**
				0,1778

Summary of Fit

RSquare	0,01846
RSquare Adj	-0,13652
Root Mean Square Error	15,48664
Mean of Response	37,05267
Observations (or Sum Wgts)	23

Analysis of Variance

Source	DF	Sum of Squares	Mean Square	F Ratio
Model	3	85,7030	28,568	0,1191
Error	19	4556,8826	239,836	**Prob > F**
C. Total	22	4642,5857		0,9478

Parameter Estimates

Term	Estimate	Std Error	t Ratio	Prob>|t|
Intercept	36,585598	3,555005	10,29	<,0001*
clasif[less60]	-2,272986	3,942873	-0,58	0,5711
gender[female]	-1,835684	4,73706	-0,39	0,7027
genetictype[black]	0,1486493	3,938318	0,04	0,9703

Effect Tests

Source	Nparm	DF	Sum of Squares	F Ratio	Prob > F
clasif	1	1	79,704384	0,3323	0,5711
gender	1	1	36,015752	0,1502	0,7027
genetictype	1	1	0,341679	0,0014	0,9703

clasif

Least Squares Means Table

Level	Least Sq Mean	Std Error	Mean
less60	34,312611	5,6113672	35,9459
S	38,858584	4,9881041	38,4914

gender

Least Squares Means Table

Level	Least Sq Mean	Std Error	Mean
female	34,749913	6,7179463	36,3098
male	38,421282	5,0024791	37,3777

genetictype

Least Squares Means Table

Level	Least Sq Mean	Std Error	Mean
black	36,734247	5,0132332	37,3228
white	36,436948	5,5825003	36,5463

Fit Group

Response 1-Nonanol

Whole Model

Effect Summary

Source	LogWorth	PValue
clasif	0,428	0,37344
genetictype	0,170	0,67610
gender	0,155	0,69985

Lack Of Fit

Source	DF	Sum of Squares	Mean Square	F Ratio
Lack Of Fit	2	73,3071	36,6536	0,5828
Pure Error	17	1069,1721	62,8925	**Prob > F**
Total Error	19	1142,4792		0,5691
				**Max RSq**
				0,1129

Summary of Fit

RSquare	0,052036
RSquare Adj	-0,09764
Root Mean Square Error	7,754385
Mean of Response	5,375417
Observations (or Sum Wgts)	23

Analysis of Variance

Source	DF	Sum of Squares	Mean Square	F Ratio
Model	3	62,7131	20,9044	0,3476
Error	19	1142,4792	60,1305	**Prob > F**
C. Total	22	1205,1923		0,7912

Parameter Estimates

Term	Estimate	Std Error	t Ratio	Prob>|t|
Intercept	4,9922076	1,780043	2,80	0,0113*
clasif[less60]	-1,799593	1,974254	-0,91	0,3734
gender[female]	-0,928375	2,371915	-0,39	0,6999
genetictype[black]	0,8367442	1,971973	0,42	0,6761

Effect Tests

Source	Nparm	DF	Sum of Squares	F Ratio	Prob > F
clasif	1	1	49,961693	0,8309	0,3734
gender	1	1	9,211774	0,1532	0,6999
genetictype	1	1	10,826253	0,1800	0,6761

clasif

Least Squares Means Table

Level	Least Sq Mean	Std Error	Mean
less60	3,1926147	2,8096933	4,42872
S	6,7918006	2,4976164	6,60613

gender

Least Squares Means Table

Level	Least Sq Mean	Std Error	Mean
female	4,0638327	3,3637736	4,99065
male	5,9205826	2,5048142	5,54375

genetictype

Least Squares Means Table

Level	Least Sq Mean	Std Error	Mean
black	5,8289519	2,5101989	5,90990
white	4,1554634	2,7952392	4,37327

Fit Group

Response 1-Nonene

Whole Model

Effect Summary

Source	LogWorth	PValue
genetictype	0,275	0,53091
clasif	0,137	0,72955
gender	0,113	0,77087

Lack Of Fit

Source	DF	Sum of Squares	Mean Square	F Ratio
Lack Of Fit	2	11,36311	5,6816	0,4060
Pure Error	17	237,87870	13,9929	**Prob > F**
Total Error	19	249,24181		0,6726
				**Max RSq**
				0,0852

Summary of Fit

RSquare	0,041512
RSquare Adj	-0,10983
Root Mean Square Error	3,621877
Mean of Response	2,521285
Observations (or Sum Wgts)	23

Analysis of Variance

Source	DF	Sum of Squares	Mean Square	F Ratio
Model	3	10,79461	3,5982	0,2743
Error	19	249,24181	13,1180	**Prob > F**
C. Total	22	260,03641		0,8432

Parameter Estimates

Term	Estimate	Std Error	t Ratio	Prob>|t|
Intercept	2,2564883	0,831413	2,71	0,0138*
clasif[less60]	-0,323539	0,922124	-0,35	0,7296
gender[female]	-0,3273	1,107861	-0,30	0,7709
genetictype[black]	0,5878897	0,921059	0,64	0,5309

Effect Tests

Source	Nparm	DF	Sum of Squares	F Ratio	Prob > F
clasif	1	1	1,6148813	0,1231	0,7296
gender	1	1	1,1449574	0,0873	0,7709
genetictype	1	1	5,3442207	0,4074	0,5309

clasif

Least Squares Means Table

Level	Least Sq Mean	Std Error	Mean
less60	1,9329498	1,3123365	2,52645
S	2,5800269	1,1665733	2,51457

gender

Least Squares Means Table

Level	Least Sq Mean	Std Error	Mean
female	1,9291880	1,5711334	1,90833
male	2,5837887	1,1699352	2,78945

genetictype

Least Squares Means Table

Level	Least Sq Mean	Std Error	Mean
black	2,8443780	1,1724503	2,97655
white	1,6685987	1,3055854	1,66766

Fit Group

Response Octanoic acid

Whole Model

Effect Summary

Source	LogWorth	PValue
genetictype	0,880	0,13188
clasif	0,072	0,84748
gender	0,013	0,97100

Lack Of Fit

Source	DF	Sum of Squares	Mean Square	F Ratio
Lack Of Fit	1	0,001054	0,001054	0,0016
Pure Error	16	10,431281	0,651955	**Prob > F**
Total Error	17	10,432335		0,9684
				**Max RSq**
				0,2006

Summary of Fit

RSquare	0,200503
RSquare Adj	0,059415
Root Mean Square Error	0,783369
Mean of Response	0,623811
Observations (or Sum Wgts)	21

Analysis of Variance

Source	DF	Sum of Squares	Mean Square	F Ratio
Model	3	2,616283	0,872094	1,4211
Error	17	10,432335	0,613667	**Prob > F**
C. Total	20	13,048618		0,2712

Parameter Estimates

Term	Estimate	Std Error	t Ratio	Prob>|t|
Intercept	0,7547906	0,208791	3,62	0,0021*
clasif[less60]	-0,0455	0,232986	-0,20	0,8475
gender[female]	0,0117373	0,318166	0,04	0,9710
genetictype[black]	-0,354994	0,224278	-1,58	0,1319

Effect Tests

Source	Nparm	DF	Sum of Squares	F Ratio	Prob > F
clasif	1	1	0,0234042	0,0381	0,8475
gender	1	1	0,0008351	0,0014	0,9710
genetictype	1	1	1,5374570	2,5054	0,1319

clasif

Least Squares Means Table

Level	Least Sq Mean	Std Error	Mean
less60	0,70929062	0,35313376	0,520056
S	0,80029057	0,26655118	0,762151

gender

Least Squares Means Table

Level	Least Sq Mean	Std Error	Mean
female	0,76652789	0,45849143	1,02502
male	0,74305330	0,28183853	0,49843

genetictype

Least Squares Means Table

Level	Least Sq Mean	Std Error	Mean
black	0,3997961	0,31452253	0,37674
white	1,1097851	0,29810117	1,11796

Fit Group

Response Tridecane

Whole Model

Effect Summary

Source	LogWorth	PValue
gender	0,428	0,37326
clasif	0,101	0,79272
genetictype	0,040	0,91132

Lack Of Fit

Source	DF	Sum of Squares	Mean Square	F Ratio
Lack Of Fit	2	138,00083	69,0004	1,6253
Pure Error	17	721,70620	42,4533	**Prob > F**
Total Error	19	859,70704		0,2260
				**Max RSq**
				0,2044

Summary of Fit

RSquare	0,052296
RSquare Adj	-0,09734
Root Mean Square Error	6,726644
Mean of Response	14,76257
Observations (or Sum Wgts)	23

Analysis of Variance

Source	DF	Sum of Squares	Mean Square	F Ratio
Model	3	47,43987	15,8133	0,3495
Error	19	859,70704	45,2477	**Prob > F**
C. Total	22	907,14691		0,7900

Parameter Estimates

Term	Estimate	Std Error	t Ratio	Prob>|t|
Intercept	14,146692	1,544122	9,16	<,0001*
clasif[less60]	-0,456407	1,712593	-0,27	0,7927
gender[female]	-1,876218	2,057549	-0,91	0,3733
genetictype[black]	-0,193069	1,710614	-0,11	0,9113

Effect Tests

Source	Nparm	DF	Sum of Squares	F Ratio	Prob > F
clasif	1	1	3,213617	0,0710	0,7927
gender	1	1	37,623834	0,8315	0,3733
genetictype	1	1	0,576394	0,0127	0,9113

clasif

Least Squares Means Table

Level	Least Sq Mean	Std Error	Mean
less60	13,690285	2,4373057	15,1739
S	14,603100	2,1665905	14,2279

gender

Least Squares Means Table

Level	Least Sq Mean	Std Error	Mean
female	12,270474	2,9179500	12,6792
male	16,022910	2,1728342	15,6740

genetictype

Least Squares Means Table

Level	Least Sq Mean	Std Error	Mean
black	13,953623	2,1775053	15,1774
white	14,339762	2,4247673	13,9848

Fit Group

Response 1-Undecene, 9-methyl-

Whole Model

Effect Summary

Source	LogWorth	PValue
clasif	0,954	0,11107
genetictype	0,026	0,94175
gender	0,012	0,97373

Lack Of Fit

Source	DF	Sum of Squares	Mean Square	F Ratio
Lack Of Fit	2	0,0299143	0,014957	0,1727
Pure Error	17	1,4720850	0,086593	**Prob > F**
Total Error	19	1,5019993		0,8428
				**Max RSq**
				0,1870

Summary of Fit

RSquare	0,170527
RSquare Adj	0,039558
Root Mean Square Error	0,281163
Mean of Response	0,63915
Observations (or Sum Wgts)	23

Analysis of Variance

Source	DF	Sum of Squares	Mean Square	F Ratio
Model	3	0,3087883	0,102929	1,3020
Error	19	1,5019993	0,079053	**Prob > F**
C. Total	22	1,8107875		0,3028

Parameter Estimates

Term	Estimate	Std Error	t Ratio	Prob>|t|
Intercept	0,6520203	0,064542	10,10	<,0001*
clasif[less60]	-0,11963	0,071584	-1,67	0,1111
gender[female]	-0,002869	0,086002	-0,03	0,9737
genetictype[black]	0,0052943	0,071501	0,07	0,9417

Effect Tests

Source	Nparm	DF	Sum of Squares	F Ratio	Prob > F
clasif	1	1	0,22078421	2,7929	0,1111
gender	1	1	0,00008799	0,0011	0,9737
genetictype	1	1	0,00043342	0,0055	0,9417

clasif

Least Squares Means Table

Level	Least Sq Mean	Std Error	Mean
less60	0,53239038	0,10187547	0,537669
S	0,77165017	0,09056001	0,771076

gender

Least Squares Means Table

Level	Least Sq Mean	Std Error	Mean
female	0,64915107	0,12196563	0,732332
male	0,65488949	0,09082099	0,598383

genetictype

Least Squares Means Table

Level	Least Sq Mean	Std Error	Mean
black	0,65731459	0,09101623	0,619542
white	0,64672597	0,10135139	0,675916

Fit Group

Response 2,4-Decadienal, (E,E)-

Whole Model

Effect Summary

Source	LogWorth		PValue
clasif	1,009		0,09801
gender	0,260		0,54967
genetictype	0,050		0,89027

Lack Of Fit

Source	DF	Sum of Squares	Mean Square	F Ratio
Lack Of Fit	2	0,553034	0,276517	0,3326
Pure Error	17	14,133156	0,831362	**Prob > F**
Total Error	19	14,686191		0,7216
				**Max RSq**
				0,1738

Summary of Fit

RSquare	0,141512
RSquare Adj	0,005961
Root Mean Square Error	0,87918
Mean of Response	1,05322
Observations (or Sum Wgts)	23

Analysis of Variance

Source	DF	Sum of Squares	Mean Square	F Ratio
Model	3	2,420846	0,806949	1,0440
Error	19	14,686191	0,772957	**Prob > F**
C. Total	22	17,107036		0,3958

Parameter Estimates

Term	Estimate	Std Error	t Ratio	Prob>|t|
Intercept	1,0304135	0,201818	5,11	<,0001*
clasif[less60]	-0,389503	0,223838	-1,74	0,0980
gender[female]	-0,163804	0,268924	-0,61	0,5497
genetictype[black]	0,0312611	0,223579	0,14	0,8903

Effect Tests

Source	Nparm	DF	Sum of Squares	F Ratio	Prob > F
clasif	1	1	2,3405061	3,0280	0,0980
gender	1	1	0,2867768	0,3710	0,5497
genetictype	1	1	0,0151112	0,0195	0,8903

clasif

Least Squares Means Table

Level	Least Sq Mean	Std Error	Mean
less60	0,6409107	0,31855861	0,79635
S	1,4199163	0,28317582	1,38716

gender

Least Squares Means Table

Level	Least Sq Mean	Std Error	Mean
female	0,8666098	0,38137936	1,13143
male	1,1942173	0,28399189	1,01900

genetictype

Least Squares Means Table

Level	Least Sq Mean	Std Error	Mean
black	1,0616746	0,28460241	1,05196
white	0,9991525	0,31691983	1,05558

Fit Group

Response 1-Tetradecanol

Whole Model

Effect Summary

Source	LogWorth	PValue
gender	0,568	0,27048
clasif	0,171	0,67466
genetictype	0,056	0,87863

Lack Of Fit

Source	DF	Sum of Squares	Mean Square	F Ratio
Lack Of Fit	2	107,10868	53,5543	2,5952
Pure Error	17	350,80714	20,6357	**Prob > F**
Total Error	19	457,91582		0,1038
				**Max RSq**
				0,2909

Summary of Fit

RSquare	0,074382
RSquare Adj	-0,07177
Root Mean Square Error	4,90926
Mean of Response	3,690965
Observations (or Sum Wgts)	23

Analysis of Variance

Source	DF	Sum of Squares	Mean Square	F Ratio
Model	3	36,79757	12,2659	0,5089
Error	19	457,91582	24,1008	**Prob > F**
C. Total	22	494,71339		0,6809

Parameter Estimates

Term	Estimate	Std Error	t Ratio	Prob>|t|
Intercept	4,2296004	1,126936	3,75	0,0013*
clasif[less60]	0,532856	1,24989	0,43	0,6747
gender[female]	1,7044204	1,501647	1,14	0,2705
genetictype[black]	0,1932272	1,248446	0,15	0,8786

Effect Tests

Source	Nparm	DF	Sum of Squares	F Ratio	Prob > F
clasif	1	1	4,380344	0,1818	0,6747
gender	1	1	31,049162	1,2883	0,2705
genetictype	1	1	0,577337	0,0240	0,8786

clasif

Least Squares Means Table

Level	Least Sq Mean	Std Error	Mean
less60	4,7624564	1,7788019	3,42430
S	3,6967445	1,5812277	4,03763

gender

Least Squares Means Table

Level	Least Sq Mean	Std Error	Mean
female	5,9340208	2,1295872	5,47060
male	2,5251800	1,5857845	2,91238

genetictype

Least Squares Means Table

Level	Least Sq Mean	Std Error	Mean
black	4,4228276	1,5891936	3,35054
white	4,0363732	1,7696511	4,32926

Fit Group

Response Phenol, 2,6-bis(1,1-dimethylethyl)-4-(1-methylpropyl)-

Whole Model

Effect Summary

Source	LogWorth	PValue
clasif	0,293	0,50905
gender	0,157	0,69737
genetictype	0,073	0,84500

Lack Of Fit

Source	DF	Sum of Squares	Mean Square	F Ratio
Lack Of Fit	2	127,50456	63,7523	1,3279
Pure Error	17	816,18765	48,0110	**Prob > F**
Total Error	19	943,69220		0,2912
				**Max RSq**
				0,2069

Summary of Fit

RSquare	0,083027
RSquare Adj	-0,06176
Root Mean Square Error	7,047554
Mean of Response	6,179677
Observations (or Sum Wgts)	23

Analysis of Variance

Source	DF	Sum of Squares	Mean Square	F Ratio
Model	3	85,4468	28,4823	0,5735
Error	19	943,6922	49,6680	**Prob > F**
C. Total	22	1029,1390		0,6394

Parameter Estimates

Term	Estimate	Std Error	t Ratio	Prob>|t|
Intercept	6,7783402	1,617788	4,19	0,0005*
clasif[less60]	-1,207558	1,794296	-0,67	0,5091
gender[female]	0,851131	2,155709	0,39	0,6974
genetictype[black]	-0,355198	1,792223	-0,20	0,8450

Effect Tests

Source	Nparm	DF	Sum of Squares	F Ratio	Prob > F
clasif	1	1	22,495974	0,4529	0,5091
gender	1	1	7,742643	0,1559	0,6974
genetictype	1	1	1,950899	0,0393	0,8450

clasif

Least Squares Means Table

Level	Least Sq Mean	Std Error	Mean
less60	5,5707819	2,5535828	4,65933
S	7,9858985	2,2699525	8,15612

gender

Least Squares Means Table

Level	Least Sq Mean	Std Error	Mean
female	7,6294712	3,0571573	8,64424
male	5,9272093	2,2764942	5,10143

genetictype

Least Squares Means Table

Level	Least Sq Mean	Std Error	Mean
black	6,4231417	2,2813881	5,39646
white	7,1335387	2,5404463	7,64821

Fit Group

Response crotal 2

Whole Model

Effect Summary

Source	LogWorth	PValue
genetictype	1,550	0,02821
gender	0,356	0,44072
clasif	0,342	0,45481

Lack Of Fit

Source	DF	Sum of Squares	Mean Square	F Ratio
Lack Of Fit	2	984,914	492,457	0,7025
Pure Error	17	11917,867	701,051	**Prob > F**
Total Error	19	12902,780		0,5092
				**Max RSq**
				0,3061

Summary of Fit

RSquare	0,248745
RSquare Adj	0,130125
Root Mean Square Error	26,05943
Mean of Response	4354,957
Observations (or Sum Wgts)	23

Analysis of Variance

Source	DF	Sum of Squares	Mean Square	F Ratio
Model	3	4272,176	1424,06	2,0970
Error	19	12902,780	679,09	**Prob > F**
C. Total	22	17174,957		0,1345

Parameter Estimates

Term	Estimate	Std Error	t Ratio	Prob>|t|
Intercept	4353,282	5,982021	727,73	<,0001*
clasif[less60]	-5,062575	6,634689	-0,76	0,4548
gender[female]	6,2769461	7,97107	0,79	0,4407
genetictype[black]	15,741916	6,627023	2,38	0,0282*

Effect Tests

Source	Nparm	DF	Sum of Squares	F Ratio	Prob > F
clasif	1	1	395,3953	0,5822	0,4548
gender	1	1	421,1078	0,6201	0,4407
genetictype	1	1	3831,8448	5,6426	0,0282*

clasif

Least Squares Means Table

Level	Least Sq Mean	Std Error	Mean
less60	4348,2195	9,4422700	4351,38
S	4358,3446	8,3935027	4359,60

gender

Least Squares Means Table

Level	Least Sq Mean	Std Error	Mean
female	4359,5590	11,304315	4356,43
male	4347,0051	8,417692	4354,31

genetictype

Least Squares Means Table

Level	Least Sq Mean	Std Error	Mean
black	4369,0240	8,4357876	4362,73
white	4337,5401	9,3936956	4340,38

Fit Group

Response C10:0

Whole Model

Effect Summary

Source	LogWorth	PValue
genetictype	0,336	0,46096
clasif	0,244	0,57031
gender	0,179	0,66273

Lack Of Fit

Source	DF	Sum of Squares	Mean Square	F Ratio
Lack Of Fit	2	0,01119245	0,005596	0,4327
Pure Error	17	0,21986286	0,012933	**Prob > F**
Total Error	19	0,23105531		0,6557
				**Max RSq**
				0,1053

Summary of Fit

RSquare	0,059757
RSquare Adj	-0,0887
Root Mean Square Error	0,110276
Mean of Response	0,080181
Observations (or Sum Wgts)	23

Analysis of Variance

Source	DF	Sum of Squares	Mean Square	F Ratio
Model	3	0,01468461	0,004895	0,4025
Error	19	0,23105531	0,012161	**Prob > F**
C. Total	22	0,24573992		0,7528

Parameter Estimates

Term	Estimate	Std Error	t Ratio	Prob>|t|
Intercept	0,0786399	0,025314	3,11	0,0058*
clasif[less60]	0,0162172	0,028076	0,58	0,5703
gender[female]	-0,014945	0,033731	-0,44	0,6627
genetictype[black]	-0,021103	0,028044	-0,75	0,4610

Effect Tests

Source	Nparm	DF	Sum of Squares	F Ratio	Prob > F
clasif	1	1	0,00405735	0,3336	0,5703
gender	1	1	0,00238719	0,1963	0,6627
genetictype	1	1	0,00688638	0,5663	0,4610

clasif

Least Squares Means Table

Level	Least Sq Mean	Std Error	Mean
less60	0,09485715	0,03995699	0,096140
S	0,06242266	0,03551890	0,059434

gender

Least Squares Means Table

Level	Least Sq Mean	Std Error	Mean
female	0,06369492	0,04783663	0,061155
male	0,09358489	0,03562126	0,088504

genetictype

Least Squares Means Table

Level	Least Sq Mean	Std Error	Mean
black	0,05753666	0,03569784	0,073902
white	0,09974315	0,03975143	0,091953

Fit Group

Response C12:0

Whole Model

Effect Summary

Source	LogWorth	PValue
clasif	1,945	0,01135
gender	0,396	0,40133
genetictype	0,268	0,53966

Lack Of Fit

Source	DF	Sum of Squares	Mean Square	F Ratio
Lack Of Fit	2	0,00092424	0,000462	0,2854
Pure Error	17	0,02752169	0,001619	**Prob > F**
Total Error	19	0,02844593		0,7552
				**Max RSq**
				0,3298

Summary of Fit

RSquare	0,307279
RSquare Adj	0,197901
Root Mean Square Error	0,038693
Mean of Response	0,100053
Observations (or Sum Wgts)	23

Analysis of Variance

Source	DF	Sum of Squares	Mean Square	F Ratio
Model	3	0,01261809	0,004206	2,8094
Error	19	0,02844593	0,001497	**Prob > F**
C. Total	22	0,04106402		0,0673

Parameter Estimates

Term	Estimate	Std Error	t Ratio	Prob>|t|
Intercept	0,1022971	0,008882	11,52	<,0001*
clasif[less60]	0,0276138	0,009851	2,80	0,0113*
gender[female]	0,0101605	0,011835	0,86	0,4013
genetictype[black]	-0,006146	0,00984	-0,62	0,5397

Effect Tests

Source	Nparm	DF	Sum of Squares	F Ratio	Prob > F
clasif	1	1	0,01176359	7,8573	0,0113*
gender	1	1	0,00110338	0,7370	0,4013
genetictype	1	1	0,00058407	0,3901	0,5397

clasif

Least Squares Means Table

Level	Least Sq Mean	Std Error	Mean
less60	0,12991083	0,01401990	0,118004
S	0,07468329	0,01246269	0,076715

gender

Least Squares Means Table

Level	Least Sq Mean	Std Error	Mean
female	0,11245753	0,01678467	0,095367
male	0,09213659	0,01249860	0,102102

genetictype

Least Squares Means Table

Level	Least Sq Mean	Std Error	Mean
black	0,09615113	0,01252547	0,097905
white	0,10844299	0,01394777	0,104080

Fit Group

Response C14:0

Whole Model

Effect Summary

Source	LogWorth	PValue
genetictype	0,937	0,11552
clasif	0,813	0,15398
gender	0,557	0,27741

Lack Of Fit

Source	DF	Sum of Squares	Mean Square	F Ratio
Lack Of Fit	2	0,05549267	0,027746	1,1706
Pure Error	17	0,40294473	0,023703	**Prob > F**
Total Error	19	0,45843740		0,3340
				**Max RSq**
				0,3073

Summary of Fit

RSquare	0,21192
RSquare Adj	0,087486
Root Mean Square Error	0,155333
Mean of Response	1,461132
Observations (or Sum Wgts)	23

Analysis of Variance

Source	DF	Sum of Squares	Mean Square	F Ratio
Model	3	0,12327680	0,041092	1,7031
Error	19	0,45843740	0,024128	**Prob > F**
C. Total	22	0,58171420		0,2002

Parameter Estimates

Term	Estimate	Std Error	t Ratio	Prob>|t|
Intercept	1,4544341	0,035657	40,79	<,0001*
clasif[less60]	0,0587235	0,039547	1,48	0,1540
gender[female]	0,0531302	0,047513	1,12	0,2774
genetictype[black]	0,0651503	0,039502	1,65	0,1155

Effect Tests

Source	Nparm	DF	Sum of Squares	F Ratio	Prob > F
clasif	1	1	0,05320007	2,2049	0,1540
gender	1	1	0,03017027	1,2504	0,2774
genetictype	1	1	0,06563354	2,7202	0,1155

clasif

Least Squares Means Table

Level	Least Sq Mean	Std Error	Mean
less60	1,5131576	0,05628268	1,50328
S	1,3957106	0,05003128	1,40634

gender

Least Squares Means Table

Level	Least Sq Mean	Std Error	Mean
female	1,5075643	0,06738180	1,43770
male	1,4013039	0,05017546	1,47138

genetictype

Least Squares Means Table

Level	Least Sq Mean	Std Error	Mean
black	1,5195844	0,05028333	1,50020
white	1,3892838	0,05599314	1,38789

Fit Group

Response C16:0

Whole Model

Effect Summary

Source	LogWorth	PValue
genetictype	1,274	0,05319
clasif	0,148	0,71094
gender	0,041	0,90925

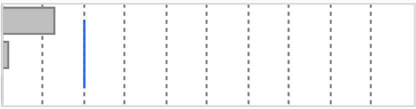

Lack Of Fit

Source	DF	Sum of Squares	Mean Square	F Ratio
Lack Of Fit	2	0,918203	0,45910	0,3586
Pure Error	17	21,763291	1,28019	**Prob > F**
Total Error	19	22,681494		0,7038
				**Max RSq**
				0,2999

Summary of Fit

RSquare	0,270356
RSquare Adj	0,155149
Root Mean Square Error	1,092595
Mean of Response	22,68874
Observations (or Sum Wgts)	23

Analysis of Variance

Source	DF	Sum of Squares	Mean Square	F Ratio
Model	3	8,404191	2,80140	2,3467
Error	19	22,681494	1,19376	**Prob > F**
C. Total	22	31,085685		0,1050

Parameter Estimates

Term	Estimate	Std Error	t Ratio	Prob>|t|
Intercept	22,485637	0,250808	89,65	<,0001*
clasif[less60]	0,104646	0,278173	0,38	0,7109
gender[female]	-0,038606	0,334203	-0,12	0,9092
genetictype[black]	0,572846	0,277851	2,06	0,0532

Effect Tests

Source	Nparm	DF	Sum of Squares	F Ratio	Prob > F
clasif	1	1	0,1689406	0,1415	0,7109
gender	1	1	0,0159297	0,0133	0,9092
genetictype	1	1	5,0742097	4,2506	0,0532

clasif

Least Squares Means Table

Level	Least Sq Mean	Std Error	Mean
less60	22,590283	0,39588641	22,9314
S	22,380991	0,35191471	22,3733

gender

Least Squares Means Table

Level	Least Sq Mean	Std Error	Mean
female	22,447030	0,47395645	22,1268
male	22,524243	0,35292887	22,9346

genetictype

Least Squares Means Table

Level	Least Sq Mean	Std Error	Mean
black	23,058482	0,35368759	23,1217
white	21,912791	0,39384983	21,8770

Fit Group

Response c9-C16:1

Whole Model

Effect Summary

Source	LogWorth	PValue
genetictype	0,438	0,36517
gender	0,353	0,44351
clasif	0,237	0,57921

Lack Of Fit

Source	DF	Sum of Squares	Mean Square	F Ratio
Lack Of Fit	2	1,488472	0,744236	1,4169
Pure Error	17	8,929204	0,525247	**Prob > F**
Total Error	19	10,417675		0,2697
				**Max RSq**
				0,2487

Summary of Fit

RSquare	0,123516
RSquare Adj	-0,01488
Root Mean Square Error	0,740472
Mean of Response	2,862935
Observations (or Sum Wgts)	23

Analysis of Variance

Source	DF	Sum of Squares	Mean Square	F Ratio
Model	3	1,468075	0,489358	0,8925
Error	19	10,417675	0,548299	**Prob > F**
C. Total	22	11,885751		0,4630

Parameter Estimates

Term	Estimate	Std Error	t Ratio	Prob>|t|
Intercept	2,7542777	0,169978	16,20	<,0001*
clasif[less60]	-0,106366	0,188523	-0,56	0,5792
gender[female]	-0,177256	0,226496	-0,78	0,4435
genetictype[black]	0,1747015	0,188305	0,93	0,3652

Effect Tests

Source	Nparm	DF	Sum of Squares	F Ratio	Prob > F
clasif	1	1	0,17453917	0,3183	0,5792
gender	1	1	0,33581221	0,6125	0,4435
genetictype	1	1	0,47193892	0,8607	0,3652

clasif

Least Squares Means Table

Level	Least Sq Mean	Std Error	Mean
less60	2,6479118	0,26829969	2,89197
S	2,8606435	0,23849924	2,82519

gender

Least Squares Means Table

Level	Least Sq Mean	Std Error	Mean
female	2,5770220	0,32120923	2,57813
male	2,9315333	0,23918656	2,98754

genetictype

Least Squares Means Table

Level	Least Sq Mean	Std Error	Mean
black	2,9289791	0,23970075	3,02351
white	2,5795762	0,26691946	2,56185

Fit Group

Response C17:0

Whole Model

Effect Summary

Source	LogWorth	PValue
gender	1,776	0,01675
genetictype	0,704	0,19785
clasif	0,336	0,46165

Lack Of Fit

Source	DF	Sum of Squares	Mean Square	F Ratio
Lack Of Fit	2	0,00237483	0,001187	0,0750
Pure Error	17	0,26902643	0,015825	**Prob > F**
Total Error	19	0,27140126		0,9280
				**Max RSq**
				0,2850

Summary of Fit

RSquare	0,278673
RSquare Adj	0,164779
Root Mean Square Error	0,119517
Mean of Response	0,322511
Observations (or Sum Wgts)	23

Analysis of Variance

Source	DF	Sum of Squares	Mean Square	F Ratio
Model	3	0,10485141	0,034950	2,4468
Error	19	0,27140126	0,014284	**Prob > F**
C. Total	22	0,37625268		0,0953

Parameter Estimates

Term	Estimate	Std Error	t Ratio	Prob>|t|
Intercept	0,3447043	0,027435	12,56	<,0001*
clasif[less60]	0,0228623	0,030429	0,75	0,4617
gender[female]	0,0958812	0,036558	2,62	0,0168*
genetictype[black]	0,0405569	0,030394	1,33	0,1979

Effect Tests

Source	Nparm	DF	Sum of Squares	F Ratio	Prob > F
clasif	1	1	0,00806360	0,5645	0,4617
gender	1	1	0,09825689	6,8787	0,0168*
genetictype	1	1	0,02543442	1,7806	0,1979

clasif

Least Squares Means Table

Level	Least Sq Mean	Std Error	Mean
less60	0,36756657	0,04330526	0,308275
S	0,32184195	0,03849528	0,341018

gender

Least Squares Means Table

Level	Least Sq Mean	Std Error	Mean
female	0,44058542	0,05184520	0,406874
male	0,24882310	0,03860622	0,285602

genetictype

Least Squares Means Table

Level	Least Sq Mean	Std Error	Mean
black	0,38526114	0,03868922	0,322569
white	0,30414738	0,04308249	0,322402

Fit Group

Response c9-C17:1

Whole Model

Effect Summary

Source	LogWorth	PValue
gender	0,999	0,10033
genetictype	0,443	0,36084
clasif	0,021	0,95210

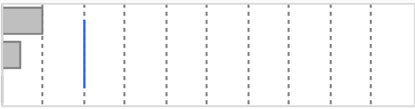

Lack Of Fit

Source	DF	Sum of Squares	Mean Square	F Ratio
Lack Of Fit	2	0,00190811	0,000954	0,1012
Pure Error	17	0,16018720	0,009423	**Prob > F**
Total Error	19	0,16209531		0,9042
				**Max RSq**
				0,1799

Summary of Fit

RSquare	0,170152
RSquare Adj	0,039124
Root Mean Square Error	0,092365
Mean of Response	0,268791
Observations (or Sum Wgts)	23

Analysis of Variance

Source	DF	Sum of Squares	Mean Square	F Ratio
Model	3	0,03323610	0,011079	1,2986
Error	19	0,16209531	0,008531	**Prob > F**
C. Total	22	0,19533141		0,3038

Parameter Estimates

Term	Estimate	Std Error	t Ratio	Prob>|t|
Intercept	0,2810066	0,021203	13,25	<,0001*
clasif[less60]	0,0014315	0,023516	0,06	0,9521
gender[female]	0,0488023	0,028253	1,73	0,1003
genetictype[black]	0,0219939	0,023489	0,94	0,3608

Effect Tests

Source	Nparm	DF	Sum of Squares	F Ratio	Prob > F
clasif	1	1	0,00003161	0,0037	0,9521
gender	1	1	0,02545520	2,9837	0,1003
genetictype	1	1	0,00747995	0,8768	0,3608

clasif

Least Squares Means Table

Level	Least Sq Mean	Std Error	Mean
less60	0,28243809	0,03346725	0,252987
S	0,27957510	0,02974999	0,289336

gender

Least Squares Means Table

Level	Least Sq Mean	Std Error	Mean
female	0,32980886	0,04006709	0,319360
male	0,23220433	0,02983572	0,246666

genetictype

Least Squares Means Table

Level	Least Sq Mean	Std Error	Mean
black	0,30300053	0,02989986	0,267689
white	0,25901266	0,03329508	0,270855

Fit Group

Response C18:0

Whole Model

Effect Summary

Source	LogWorth	PValue
clasif	1,023	0,09487
gender	0,925	0,11873
genetictype	0,178	0,66366

Lack Of Fit

Source	DF	Sum of Squares	Mean Square	F Ratio
Lack Of Fit	2	0,043021	0,021511	0,0267
Pure Error	17	13,700021	0,805884	**Prob > F**
Total Error	19	13,743042		0,9737
				**Max RSq**
				0,2077

Summary of Fit

RSquare	0,205251
RSquare Adj	0,079764
Root Mean Square Error	0,850481
Mean of Response	11,71396
Observations (or Sum Wgts)	23

Analysis of Variance

Source	DF	Sum of Squares	Mean Square	F Ratio
Model	3	3,549262	1,18309	1,6356
Error	19	13,743042	0,72332	**Prob > F**
C. Total	22	17,292304		0,2145

Parameter Estimates

Term	Estimate	Std Error	t Ratio	Prob>|t|
Intercept	11,859714	0,195231	60,75	<,0001*
clasif[less60]	0,3806305	0,216531	1,76	0,0949
gender[female]	0,4250593	0,260146	1,63	0,1187
genetictype[black]	-0,09554	0,216281	-0,44	0,6637

Effect Tests

Source	Nparm	DF	Sum of Squares	F Ratio	Prob > F
clasif	1	1	2,2350939	3,0901	0,0949
gender	1	1	1,9310585	2,6697	0,1187
genetictype	1	1	0,1411450	0,1951	0,6637

clasif

Least Squares Means Table

Level	Least Sq Mean	Std Error	Mean
less60	12,240344	0,30815996	11,8292
S	11,479083	0,27393216	11,5641

gender

Least Squares Means Table

Level	Least Sq Mean	Std Error	Mean
female	12,284773	0,36893006	12,0538
male	11,434654	0,27472159	11,5653

genetictype

Least Squares Means Table

Level	Least Sq Mean	Std Error	Mean
black	11,764173	0,27531218	11,5793
white	11,955254	0,30657467	11,9664

Fit Group

Response c9-C18:1

Whole Model

Effect Summary

Source	LogWorth	PValue
gender	1,649	0,02243
clasif	1,181	0,06595
genetictype	0,139	0,72554

Lack Of Fit

Source	DF	Sum of Squares	Mean Square	F Ratio
Lack Of Fit	2	8,90751	4,45376	0,5970
Pure Error	17	126,83259	7,46074	**Prob > F**
Total Error	19	135,74010		0,5616
				**Max RSq**
				0,3646

Summary of Fit

RSquare	0,319972
RSquare Adj	0,212599
Root Mean Square Error	2,672867
Mean of Response	38,45095
Observations (or Sum Wgts)	23

Analysis of Variance

Source	DF	Sum of Squares	Mean Square	F Ratio
Model	3	63,86940	21,2898	2,9800
Error	19	135,74010	7,1442	**Prob > F**
C. Total	22	199,60950		0,0573

Parameter Estimates

Term	Estimate	Std Error	t Ratio	Prob>|t|
Intercept	37,755364	0,613565	61,53	<,0001*
clasif[less60]	-1,327742	0,680508	-1,95	0,0659
gender[female]	-2,031824	0,817578	-2,49	0,0224*
genetictype[black]	0,2421837	0,679721	0,36	0,7255

Effect Tests

Source	Nparm	DF	Sum of Squares	F Ratio	Prob > F
clasif	1	1	27,196679	3,8068	0,0659
gender	1	1	44,123345	6,1761	0,0224*
genetictype	1	1	0,906948	0,1269	0,7255

clasif

Least Squares Means Table

Level	Least Sq Mean	Std Error	Mean
less60	36,427622	0,96847597	38,2773
S	39,083105	0,86090587	38,6767

gender

Least Squares Means Table

Level	Least Sq Mean	Std Error	Mean
female	35,723540	1,1594625	36,5681
male	39,787187	0,8633869	39,2747

genetictype

Least Squares Means Table

Level	Least Sq Mean	Std Error	Mean
black	37,997547	0,86524295	39,0450
white	37,513180	0,96349378	37,3372

Fit Group

Response c11-C18:1

Whole Model

Effect Summary

Source	LogWorth	PValue
gender	0,596	0,25361
clasif	0,423	0,37763
genetictype	0,184	0,65536

Lack Of Fit

Source	DF	Sum of Squares	Mean Square	F Ratio
Lack Of Fit	2	0,8715612	0,435781	1,1921
Pure Error	17	6,2144065	0,365553	**Prob > F**
Total Error	19	7,0859678		0,3277
				**Max RSq**
				0,2275

Summary of Fit

RSquare	0,11914
RSquare Adj	-0,01994
Root Mean Square Error	0,610693
Mean of Response	3,112355
Observations (or Sum Wgts)	23

Analysis of Variance

Source	DF	Sum of Squares	Mean Square	F Ratio
Model	3	0,9584086	0,319470	0,8566
Error	19	7,0859678	0,372946	**Prob > F**
C. Total	22	8,0443764		0,4804

Parameter Estimates

Term	Estimate	Std Error	t Ratio	Prob>|t|
Intercept	3,0231873	0,140186	21,57	<,0001*
clasif[less60]	-0,140459	0,155481	-0,90	0,3776
gender[female]	-0,219921	0,186799	-1,18	0,2536
genetictype[black]	0,0704227	0,155302	0,45	0,6554

Effect Tests

Source	Nparm	DF	Sum of Squares	F Ratio	Prob > F
clasif	1	1	0,30436206	0,8161	0,3776
gender	1	1	0,51692726	1,3861	0,2536
genetictype	1	1	0,07668633	0,2056	0,6554

clasif

Least Squares Means Table

Level	Least Sq Mean	Std Error	Mean
less60	2,8827278	0,22127602	3,10673
S	3,1636468	0,19669856	3,11966

gender

Least Squares Means Table

Level	Least Sq Mean	Std Error	Mean
female	2,8032664	0,26491234	2,87341
male	3,2431082	0,19726541	3,21689

genetictype

Least Squares Means Table

Level	Least Sq Mean	Std Error	Mean
black	3,0936100	0,19768949	3,20807
white	2,9527646	0,22013770	2,93290

Fit Group

Response c9,12-C18:2

Whole Model

Effect Summary

Source	LogWorth	PValue
gender	0,914	0,12195
clasif	0,435	0,36729
genetictype	0,390	0,40780

Lack Of Fit

Source	DF	Sum of Squares	Mean Square	F Ratio
Lack Of Fit	2	30,03862	15,0193	1,2838
Pure Error	17	198,88576	11,6992	**Prob > F**
Total Error	19	228,92438		0,3025
				**Max RSq**
				0,3347

Summary of Fit

RSquare	0,23421
RSquare Adj	0,113295
Root Mean Square Error	3,471117
Mean of Response	14,81372
Observations (or Sum Wgts)	23

Analysis of Variance

Source	DF	Sum of Squares	Mean Square	F Ratio
Model	3	70,01433	23,3381	1,9370
Error	19	228,92438	12,0487	**Prob > F**
C. Total	22	298,93871		0,1579

Parameter Estimates

Term	Estimate	Std Error	t Ratio	Prob>|t|
Intercept	15,607287	0,796805	19,59	<,0001*
clasif[less60]	0,8162003	0,883741	0,92	0,3673
gender[female]	1,7188788	1,061747	1,62	0,1219
genetictype[black]	-0,747235	0,88272	-0,85	0,4078

Effect Tests

Source	Nparm	DF	Sum of Squares	F Ratio	Prob > F
clasif	1	1	10,277372	0,8530	0,3673
gender	1	1	31,578170	2,6209	0,1219
genetictype	1	1	8,633912	0,7166	0,4078

clasif

Least Squares Means Table

Level	Least Sq Mean	Std Error	Mean
less60	16,423487	1,2577108	14,5667
S	14,791087	1,1180149	15,1349

gender

Least Squares Means Table

Level	Least Sq Mean	Std Error	Mean
female	17,326166	1,5057353	17,0634
male	13,888408	1,1212369	13,8295

genetictype

Least Squares Means Table

Level	Least Sq Mean	Std Error	Mean
black	14,860052	1,1236473	13,8716
white	16,354522	1,2512407	16,5802

Fit Group

Response C18:3 n6

Whole Model

Effect Summary

Source	LogWorth	PValue
gender	0,701	0,19893
genetictype	0,157	0,69612
clasif	0,087	0,81806

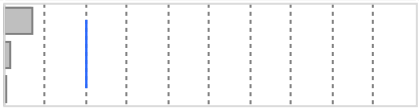

Lack Of Fit

Source	DF	Sum of Squares	Mean Square	F Ratio
Lack Of Fit	2	0,00054402	0,000272	0,1220
Pure Error	17	0,03790147	0,002229	**Prob > F**
Total Error	19	0,03844549		0,8859
				**Max RSq**
				0,2198

Summary of Fit

RSquare	0,208597
RSquare Adj	0,083638
Root Mean Square Error	0,044983
Mean of Response	0,09442
Observations (or Sum Wgts)	23

Analysis of Variance

Source	DF	Sum of Squares	Mean Square	F Ratio
Model	3	0,01013339	0,003378	1,6693
Error	19	0,03844549	0,002023	**Prob > F**
C. Total	22	0,04857888		0,2072

Parameter Estimates

Term	Estimate	Std Error	t Ratio	Prob>|t|
Intercept	0,1033149	0,010326	10,01	<,0001*
clasif[less60]	-0,002671	0,011453	-0,23	0,8181
gender[female]	0,018314	0,013759	1,33	0,1989
genetictype[black]	-0,004536	0,011439	-0,40	0,6961

Effect Tests

Source	Nparm	DF	Sum of Squares	F Ratio	Prob > F
clasif	1	1	0,00011009	0,0544	0,8181
gender	1	1	0,00358479	1,7716	0,1989
genetictype	1	1	0,00031819	0,1573	0,6961

clasif

Least Squares Means Table

Level	Least Sq Mean	Std Error	Mean
less60	0,10064360	0,01629887	0,082705
S	0,10598621	0,01448853	0,109649

gender

Least Squares Means Table

Level	Least Sq Mean	Std Error	Mean
female	0,12162892	0,01951306	0,125481
male	0,08500088	0,01453028	0,080830

genetictype

Least Squares Means Table

Level	Least Sq Mean	Std Error	Mean
black	0,09877863	0,01456152	0,084458
white	0,10785117	0,01621502	0,113098

Fit Group

Response C18:3 n3

Whole Model

Effect Summary

Source	LogWorth	PValue
gender	0,831	0,14766
clasif	0,584	0,26061
genetictype	0,317	0,48177

Lack Of Fit

Source	DF	Sum of Squares	Mean Square	F Ratio
Lack Of Fit	2	0,3091099	0,154555	1,4304
Pure Error	17	1,8368232	0,108048	**Prob > F**
Total Error	19	2,1459331		0,2666
				**Max RSq**
				0,3128

Summary of Fit

RSquare	0,197174
RSquare Adj	0,070413
Root Mean Square Error	0,336071
Mean of Response	1,152019
Observations (or Sum Wgts)	23

Analysis of Variance

Source	DF	Sum of Squares	Mean Square	F Ratio
Model	3	0,5270426	0,175681	1,5555
Error	19	2,1459331	0,112944	**Prob > F**
C. Total	22	2,6729757		0,2329

Parameter Estimates

Term	Estimate	Std Error	t Ratio	Prob>|t|
Intercept	1,2184562	0,077146	15,79	<,0001*
clasif[less60]	0,0992112	0,085563	1,16	0,2606
gender[female]	0,1551576	0,102798	1,51	0,1477
genetictype[black]	-0,061324	0,085464	-0,72	0,4818

Effect Tests

Source	Nparm	DF	Sum of Squares	F Ratio	Prob > F
clasif	1	1	0,15184823	1,3445	0,2606
gender	1	1	0,25730152	2,2781	0,1477
genetictype	1	1	0,05815034	0,5149	0,4818

clasif

Least Squares Means Table

Level	Least Sq Mean	Std Error	Mean
less60	1,3176674	0,12177072	1,15336
S	1,1192450	0,10824546	1,15028

gender

Least Squares Means Table

Level	Least Sq Mean	Std Error	Mean
female	1,3736138	0,14578428	1,32903
male	1,0632986	0,10855740	1,07458

genetictype

Least Squares Means Table

Level	Least Sq Mean	Std Error	Mean
black	1,1571323	0,10879078	1,07642
white	1,2797801	0,12114428	1,29377

Fit Group

Response C20:0

Whole Model

Effect Summary

Source	LogWorth	PValue
gender	0,738	0,18262
genetictype	0,321	0,47730
clasif	0,124	0,75083

Lack Of Fit

Source	DF	Sum of Squares	Mean Square	F Ratio
Lack Of Fit	2	0,00432825	0,002164	2,1038
Pure Error	17	0,01748720	0,001029	**Prob > F**
Total Error	19	0,02181545		0,1526
				**Max RSq**
				0,4132

Summary of Fit

RSquare	0,267951
RSquare Adj	0,152364
Root Mean Square Error	0,033885
Mean of Response	0,108645
Observations (or Sum Wgts)	23

Analysis of Variance

Source	DF	Sum of Squares	Mean Square	F Ratio
Model	3	0,00798507	0,002662	2,3182
Error	19	0,02181545	0,001148	**Prob > F**
C. Total	22	0,02980053		0,1080

Parameter Estimates

Term	Estimate	Std Error	t Ratio	Prob>|t|
Intercept	0,1007706	0,007778	12,96	<,0001*
clasif[less60]	0,0027796	0,008627	0,32	0,7508
gender[female]	-0,014337	0,010365	-1,38	0,1826
genetictype[black]	0,0062472	0,008617	0,72	0,4773

Effect Tests

Source	Nparm	DF	Sum of Squares	F Ratio	Prob > F
clasif	1	1	0,00011919	0,1038	0,7508
gender	1	1	0,00219699	1,9134	0,1826
genetictype	1	1	0,00060348	0,5256	0,4773

clasif

Least Squares Means Table

Level	Least Sq Mean	Std Error	Mean
less60	0,10355013	0,01227770	0,119046
S	0,09799103	0,01091399	0,095124

gender

Least Squares Means Table

Level	Least Sq Mean	Std Error	Mean
female	0,08643334	0,01469890	0,081771
male	0,11510782	0,01094545	0,120402

genetictype

Least Squares Means Table

Level	Least Sq Mean	Std Error	Mean
black	0,10701776	0,01096898	0,118458
white	0,09452340	0,01221454	0,090244

Fit Group

Response c11-C20:1

Whole Model

Effect Summary

Source	LogWorth	PValue
gender	1,953	0,01115
clasif	0,179	0,66205
genetictype	0,107	0,78182

Lack Of Fit

Source	DF	Sum of Squares	Mean Square	F Ratio
Lack Of Fit	2	0,00511254	0,002556	0,5598
Pure Error	17	0,07762904	0,004566	**Prob > F**
Total Error	19	0,08274158		0,5815
				**Max RSq**
				0,4124

Summary of Fit

RSquare	0,373706
RSquare Adj	0,274817
Root Mean Square Error	0,065991
Mean of Response	0,58862
Observations (or Sum Wgts)	23

Analysis of Variance

Source	DF	Sum of Squares	Mean Square	F Ratio
Model	3	0,04937143	0,016457	3,7791
Error	19	0,08274158	0,004355	**Prob > F**
C. Total	22	0,13211301		0,0279*

Parameter Estimates

Term	Estimate	Std Error	t Ratio	Prob>|t|
Intercept	0,5688228	0,015148	37,55	<,0001*
clasif[less60]	-0,00746	0,016801	-0,44	0,6621
gender[female]	-0,056747	0,020185	-2,81	0,0111*
genetictype[black]	-0,004714	0,016782	-0,28	0,7818

Effect Tests

Source	Nparm	DF	Sum of Squares	F Ratio	Prob > F
clasif	1	1	0,00085851	0,1971	0,6621
gender	1	1	0,03441736	7,9033	0,0111*
genetictype	1	1	0,00034364	0,0789	0,7818

clasif

Least Squares Means Table

Level	Least Sq Mean	Std Error	Mean
less60	0,56136300	0,02391094	0,606841
S	0,57628263	0,02125512	0,564933

gender

Least Squares Means Table

Level	Least Sq Mean	Std Error	Mean
female	0,51207612	0,02862625	0,519425
male	0,62556951	0,02131637	0,618893

genetictype

Least Squares Means Table

Level	Least Sq Mean	Std Error	Mean
black	0,56410864	0,02136220	0,603236
white	0,57353699	0,02378794	0,561215

Fit Group

Response C20:2

Whole Model

Effect Summary

Source	LogWorth	PValue
genetictype	1,046	0,08998
clasif	0,397	0,40050
gender	0,226	0,59456

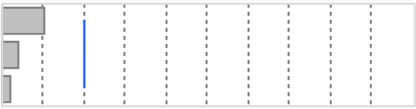

Lack Of Fit

Source	DF	Sum of Squares	Mean Square	F Ratio
Lack Of Fit	2	0,04333230	0,021666	1,6463
Pure Error	17	0,22372890	0,013161	**Prob > F**
Total Error	19	0,26706119		0,2220
				**Max RSq**
				0,3477

Summary of Fit

RSquare	0,221344
RSquare Adj	0,098398
Root Mean Square Error	0,118557
Mean of Response	0,481861
Observations (or Sum Wgts)	23

Analysis of Variance

Source	DF	Sum of Squares	Mean Square	F Ratio
Model	3	0,07591579	0,025305	1,8003
Error	19	0,26706119	0,014056	**Prob > F**
C. Total	22	0,34297698		0,1813

Parameter Estimates

Term	Estimate	Std Error	t Ratio	Prob>|t|
Intercept	0,5025501	0,027215	18,47	<,0001*
clasif[less60]	0,0259595	0,030185	0,86	0,4005
gender[female]	0,0196318	0,036264	0,54	0,5946
genetictype[black]	-0,053864	0,03015	-1,79	0,0900

Effect Tests

Source	Nparm	DF	Sum of Squares	F Ratio	Prob > F
clasif	1	1	0,01039632	0,7396	0,4005
gender	1	1	0,00411922	0,2931	0,5946
genetictype	1	1	0,04486258	3,1917	0,0900

clasif

Least Squares Means Table

Level	Least Sq Mean	Std Error	Mean
less60	0,52850959	0,04295761	0,482895
S	0,47659068	0,03818625	0,480517

gender

Least Squares Means Table

Level	Least Sq Mean	Std Error	Mean
female	0,52218189	0,05142899	0,526724
male	0,48291838	0,03829629	0,462233

genetictype

Least Squares Means Table

Level	Least Sq Mean	Std Error	Mean
black	0,44868648	0,03837862	0,442943
white	0,55641379	0,04273663	0,554832

Fit Group

Response C20:3 n6

Whole Model

Effect Summary

Source	LogWorth		PValue
genetictype	1,005		0,09890
clasif	0,089		0,81489
gender	0,028		0,93790

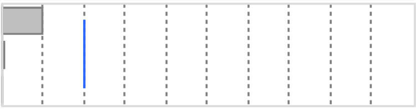

Lack Of Fit

Source	DF	Sum of Squares	Mean Square	F Ratio
Lack Of Fit	2	0,00226528	0,001133	0,2076
Pure Error	17	0,09273926	0,005455	**Prob > F**
Total Error	19	0,09500454		0,8145
				**Max RSq**
				0,2016

Summary of Fit

RSquare	0,182054
RSquare Adj	0,052905
Root Mean Square Error	0,070712
Mean of Response	0,212581
Observations (or Sum Wgts)	23

Analysis of Variance

Source	DF	Sum of Squares	Mean Square	F Ratio
Model	3	0,02114566	0,007049	1,4096
Error	19	0,09500454	0,005000	**Prob > F**
C. Total	22	0,11615020		0,2708

Parameter Estimates

Term	Estimate	Std Error	t Ratio	Prob>|t|
Intercept	0,2219663	0,016232	13,67	<,0001*
clasif[less60]	-0,004274	0,018003	-0,24	0,8149
gender[female]	-0,001708	0,02163	-0,08	0,9379
genetictype[black]	-0,031202	0,017982	-1,74	0,0989

Effect Tests

Source	Nparm	DF	Sum of Squares	F Ratio	Prob > F
clasif	1	1	0,00028180	0,0564	0,8149
gender	1	1	0,00003117	0,0062	0,9379
genetictype	1	1	0,01505465	3,0108	0,0989

clasif

Least Squares Means Table

Level	Least Sq Mean	Std Error	Mean
less60	0,21769236	0,02562164	0,202336
S	0,22624024	0,02277581	0,225899

gender

Least Squares Means Table

Level	Least Sq Mean	Std Error	Mean
female	0,22025862	0,03067431	0,236684
male	0,22367398	0,02284144	0,202035

genetictype

Least Squares Means Table

Level	Least Sq Mean	Std Error	Mean
black	0,19076382	0,02289055	0,190591
white	0,25316877	0,02548983	0,253810

Fit Group

Response C20:4

Whole Model

Effect Summary

Source	LogWorth	PValue
genetictype	0,770	0,16986
gender	0,045	0,90112
clasif	0,001	0,99710

Lack Of Fit

Source	DF	Sum of Squares	Mean Square	F Ratio
Lack Of Fit	2	0,0674123	0,033706	0,1667
Pure Error	17	3,4377678	0,202222	**Prob > F**
Total Error	19	3,5051801		0,8478
				**Max RSq**
				0,1327

Summary of Fit

RSquare	0,11572
RSquare Adj	-0,0239
Root Mean Square Error	0,429515
Mean of Response	1,317391
Observations (or Sum Wgts)	23

Analysis of Variance

Source	DF	Sum of Squares	Mean Square	F Ratio
Model	3	0,4586985	0,152899	0,8288
Error	19	3,5051801	0,184483	**Prob > F**
C. Total	22	3,9638786		0,4943

Parameter Estimates

Term	Estimate	Std Error	t Ratio	Prob>|t|
Intercept	1,3582969	0,098596	13,78	<,0001*
clasif[less60]	0,0004031	0,109354	0,00	0,9971
gender[female]	-0,016543	0,13138	-0,13	0,9011
genetictype[black]	-0,155846	0,109228	-1,43	0,1699

Effect Tests

Source	Nparm	DF	Sum of Squares	F Ratio	Prob > F
clasif	1	1	0,00000251	0,0000	0,9971
gender	1	1	0,00292497	0,0159	0,9011
genetictype	1	1	0,37556397	2,0358	0,1699

clasif

Least Squares Means Table

Level	Least Sq Mean	Std Error	Mean
less60	1,3587000	0,15562879	1,28878
S	1,3578938	0,13834286	1,35459

gender

Least Squares Means Table

Level	Least Sq Mean	Std Error	Mean
female	1,3417540	0,18631927	1,40826
male	1,3748399	0,13874155	1,27764

genetictype

Least Squares Means Table

Level	Least Sq Mean	Std Error	Mean
black	1,2024510	0,13903981	1,21472
white	1,5141429	0,15482818	1,50991

Fit Group

Response C20:3 n3

Whole Model

Effect Summary

Source	LogWorth	PValue
clasif	1,320	0,04791
gender	0,523	0,29970
genetictype	0,280	0,52526

Lack Of Fit

Source	DF	Sum of Squares	Mean Square	F Ratio
Lack Of Fit	2	0,01093735	0,005469	1,8457
Pure Error	17	0,05036889	0,002963	**Prob > F**
Total Error	19	0,06130624		0,1882
				**Max RSq**
				0,3512

Summary of Fit

RSquare	0,210309
RSquare Adj	0,085621
Root Mean Square Error	0,056804
Mean of Response	0,139142
Observations (or Sum Wgts)	23

Analysis of Variance

Source	DF	Sum of Squares	Mean Square	F Ratio
Model	3	0,01632700	0,005442	1,6867
Error	19	0,06130624	0,003227	**Prob > F**
C. Total	22	0,07763324		0,2036

Parameter Estimates

Term	Estimate	Std Error	t Ratio	Prob>|t|
Intercept	0,145247	0,013039	11,14	<,0001*
clasif[less60]	0,0305818	0,014462	2,11	0,0479*
gender[female]	0,018525	0,017375	1,07	0,2997
genetictype[black]	-0,009349	0,014445	-0,65	0,5253

Effect Tests

Source	Nparm	DF	Sum of Squares	F Ratio	Prob > F
clasif	1	1	0,01442826	4,4716	0,0479*
gender	1	1	0,00366784	1,1367	0,2997
genetictype	1	1	0,00135142	0,4188	0,5253

clasif

Least Squares Means Table

Level	Least Sq Mean	Std Error	Mean
less60	0,17582872	0,02058198	0,155120
S	0,11466518	0,01829591	0,118370

gender

Least Squares Means Table

Level	Least Sq Mean	Std Error	Mean
female	0,16377191	0,02464081	0,145934
male	0,12672199	0,01834863	0,136170

genetictype

Least Squares Means Table

Level	Least Sq Mean	Std Error	Mean
black	0,13589829	0,01838808	0,132507
white	0,15459561	0,02047610	0,151581

Fit Group

Response PUFA

Whole Model

Effect Summary

Source	LogWorth	PValue
gender	0,855	0,13952
genetictype	0,501	0,31526
clasif	0,442	0,36132

Lack Of Fit

Source	DF	Sum of Squares	Mean Square	F Ratio
Lack Of Fit	2	37,01418	18,5071	1,1432
Pure Error	17	275,20799	16,1887	**Prob > F**
Total Error	19	312,22217		0,3421
				**Max RSq**
				0,3347

Summary of Fit

RSquare	0,245267
RSquare Adj	0,126098
Root Mean Square Error	4,053732
Mean of Response	18,21114
Observations (or Sum Wgts)	23

Analysis of Variance

Source	DF	Sum of Squares	Mean Square	F Ratio
Model	3	101,46321	33,8211	2,0582
Error	19	312,22217	16,4327	**Prob > F**
C. Total	22	413,68538		0,1398

Parameter Estimates

Term	Estimate	Std Error	t Ratio	Prob>|t|
Intercept	19,157118	0,930547	20,59	<,0001*
clasif[less60]	0,9654106	1,032074	0,94	0,3613
gender[female]	1,9122565	1,239958	1,54	0,1395
genetictype[black]	-1,063356	1,030881	-1,03	0,3153

Effect Tests

Source	Nparm	DF	Sum of Squares	F Ratio	Prob > F
clasif	1	1	14,378470	0,8750	0,3613
gender	1	1	39,083075	2,3784	0,1395
genetictype	1	1	17,484386	1,0640	0,3153

clasif

Least Squares Means Table

Level	Least Sq Mean	Std Error	Mean
less60	20,122529	1,4688134	17,9319
S	18,191708	1,3056701	18,5742

gender

Least Squares Means Table

Level	Least Sq Mean	Std Error	Mean
female	21,069375	1,7584680	20,8355
male	17,244862	1,3094328	17,0630

genetictype

Least Squares Means Table

Level	Least Sq Mean	Std Error	Mean
black	18,093762	1,3122478	17,0132
white	20,220475	1,4612573	20,4572

Fit Group

Response MUFA

Whole Model

Effect Summary

Source	LogWorth	PValue
gender	1,404	0,03947
clasif	0,995	0,10110
genetictype	0,230	0,58823

Lack Of Fit

Source	DF	Sum of Squares	Mean Square	F Ratio
Lack Of Fit	2	25,68724	12,8436	0,9883
Pure Error	17	220,93446	12,9961	**Prob > F**
Total Error	19	246,62170		0,3926
				**Max RSq**
				0,3661

Summary of Fit

RSquare	0,292364
RSquare Adj	0,180632
Root Mean Square Error	3,602789
Mean of Response	45,28365
Observations (or Sum Wgts)	23

Analysis of Variance

Source	DF	Sum of Squares	Mean Square	F Ratio
Model	3	101,89330	33,9644	2,6167
Error	19	246,62170	12,9801	**Prob > F**
C. Total	22	348,51500		0,0808

Parameter Estimates

Term	Estimate	Std Error	t Ratio	Prob>|t|
Intercept	44,382658	0,827031	53,67	<,0001*
clasif[less60]	-1,580595	0,917264	-1,72	0,1011
gender[female]	-2,436944	1,102023	-2,21	0,0395*
genetictype[black]	0,5045876	0,916205	0,55	0,5882

Effect Tests

Source	Nparm	DF	Sum of Squares	F Ratio	Prob > F
clasif	1	1	38,541627	2,9693	0,1011
gender	1	1	63,472809	4,8900	0,0395*
genetictype	1	1	3,937004	0,3033	0,5882

clasif

Least Squares Means Table

Level	Least Sq Mean	Std Error	Mean
less60	42,802062	1,3054204	45,1358
S	45,963253	1,1604254	45,4759

gender

Least Squares Means Table

Level	Least Sq Mean	Std Error	Mean
female	41,945713	1,5628534	42,8585
male	46,819602	1,1637696	46,3447

genetictype

Least Squares Means Table

Level	Least Sq Mean	Std Error	Mean
black	44,887246	1,1662714	46,1475
white	43,878070	1,2987049	43,6640

Fit Group

Response SFA

Whole Model

Effect Summary

Source	LogWorth	PValue
clasif	1,219	0,06043
genetictype	1,082	0,08283
gender	0,749	0,17805

Lack Of Fit

Source	DF	Sum of Squares	Mean Square	F Ratio
Lack Of Fit	2	1,030735	0,51537	0,3288
Pure Error	17	26,648951	1,56759	**Prob > F**
Total Error	19	27,679686		0,7243
				**Max RSq**
				0,3118

Summary of Fit

RSquare	0,285193
RSquare Adj	0,172329
Root Mean Square Error	1,20699
Mean of Response	36,47521
Observations (or Sum Wgts)	23

Analysis of Variance

Source	DF	Sum of Squares	Mean Square	F Ratio
Model	3	11,043616	3,68121	2,5269
Error	19	27,679686	1,45683	**Prob > F**
C. Total	22	38,723302		0,0881

Parameter Estimates

Term	Estimate	Std Error	t Ratio	Prob>|t|
Intercept	36,426196	0,277068	131,47	<,0001*
clasif[less60]	0,6134729	0,307298	2,00	0,0604
gender[female]	0,5163428	0,369195	1,40	0,1781
genetictype[black]	0,5620109	0,306943	1,83	0,0828

Effect Tests

Source	Nparm	DF	Sum of Squares	F Ratio	Prob > F
clasif	1	1	5,8060308	3,9854	0,0604
gender	1	1	2,8495265	1,9560	0,1781
genetictype	1	1	4,8840736	3,3525	0,0828

clasif

Least Squares Means Table

Level	Least Sq Mean	Std Error	Mean
less60	37,039669	0,43733612	36,9054
S	35,812723	0,38876053	35,9160

gender

Least Squares Means Table

Level	Least Sq Mean	Std Error	Mean
female	36,942539	0,52358017	36,2635
male	35,909853	0,38988088	36,5678

genetictype

Least Squares Means Table

Level	Least Sq Mean	Std Error	Mean
black	36,988207	0,39071904	36,8140
white	35,864185	0,43508631	35,8399

Fit Group

Response PUFA/SFA

Whole Model

Effect Summary

Source	LogWorth	PValue
genetictype	0,626	0,23636
gender	0,624	0,23753
clasif	0,272	0,53490

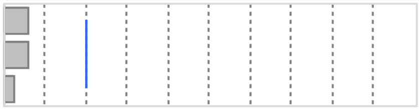

Lack Of Fit

Source	DF	Sum of Squares	Mean Square	F Ratio
Lack Of Fit	2	0,03117867	0,015589	1,0752
Pure Error	17	0,24648038	0,014499	**Prob > F**
Total Error	19	0,27765905		0,3633
				**Max RSq**
				0,3162

Summary of Fit

RSquare	0,229709
RSquare Adj	0,108084
Root Mean Square Error	0,120887
Mean of Response	0,501636
Observations (or Sum Wgts)	23

Analysis of Variance

Source	DF	Sum of Squares	Mean Square	F Ratio
Model	3	0,08280092	0,027600	1,8887
Error	19	0,27765905	0,014614	**Prob > F**
C. Total	22	0,36045998		0,1658

Parameter Estimates

Term	Estimate	Std Error	t Ratio	Prob>|t|
Intercept	0,5281865	0,02775	19,03	<,0001*
clasif[less60]	0,0194523	0,030778	0,63	0,5349
gender[female]	0,0450984	0,036977	1,22	0,2375
genetictype[black]	-0,037591	0,030742	-1,22	0,2364

Effect Tests

Source	Nparm	DF	Sum of Squares	F Ratio	Prob > F
clasif	1	1	0,00583752	0,3995	0,5349
gender	1	1	0,02173791	1,4875	0,2375
genetictype	1	1	0,02185060	1,4952	0,2364

clasif

Least Squares Means Table

Level	Least Sq Mean	Std Error	Mean
less60	0,54763876	0,04380167	0,489237
S	0,50873424	0,03893655	0,517754

gender

Least Squares Means Table

Level	Least Sq Mean	Std Error	Mean
female	0,57328486	0,05243950	0,575501
male	0,48308815	0,03904876	0,469320

genetictype

Least Squares Means Table

Level	Least Sq Mean	Std Error	Mean
black	0,49059537	0,03913271	0,464007
white	0,56577763	0,04357634	0,572189

Fit Group

Response MUFA/SFA

Whole Model

Effect Summary

Source	LogWorth	PValue
gender	1,919	0,01206
clasif	1,719	0,01911
genetictype	0,056	0,87934

Lack Of Fit

Source	DF	Sum of Squares	Mean Square	F Ratio
Lack Of Fit	2	0,01132167	0,005661	0,5632
Pure Error	17	0,17086694	0,010051	**Prob > F**
Total Error	19	0,18218860		0,5796
				**Max RSq**
				0,3901

Summary of Fit

RSquare	0,349675
RSquare Adj	0,246993
Root Mean Square Error	0,097923
Mean of Response	1,2426
Observations (or Sum Wgts)	23

Analysis of Variance

Source	DF	Sum of Squares	Mean Square	F Ratio
Model	3	0,09796166	0,032654	3,4054
Error	19	0,18218860	0,009589	**Prob > F**
C. Total	22	0,28015027		0,0388*

Parameter Estimates

Term	Estimate	Std Error	t Ratio	Prob>|t|
Intercept	1,2195695	0,022478	54,25	<,0001*
clasif[less60]	-0,063847	0,024931	-2,56	0,0191*
gender[female]	-0,083118	0,029953	-2,77	0,0121*
genetictype[black]	-0,003832	0,024902	-0,15	0,8793

Effect Tests

Source	Nparm	DF	Sum of Squares	F Ratio	Prob > F
clasif	1	1	0,06288819	6,5585	0,0191*
gender	1	1	0,07383969	7,7006	0,0121*
genetictype	1	1	0,00022702	0,0237	0,8793

clasif

Least Squares Means Table

Level	Least Sq Mean	Std Error	Mean
less60	1,1557225	0,03548096	1,22399
S	1,2834165	0,03154004	1,26679

gender

Least Squares Means Table

Level	Least Sq Mean	Std Error	Mean
female	1,1364512	0,04247792	1,18370
male	1,3026878	0,03163093	1,26837

genetictype

Least Squares Means Table

Level	Least Sq Mean	Std Error	Mean
black	1,2157378	0,03169893	1,25541
white	1,2234012	0,03529843	1,21858
